# Electrode/Electrolyte Optimization-Induced Double-Layered Architecture for High-Performance Aqueous Zinc-(Dual) Halogen Batteries

**DOI:** 10.1007/s40820-024-01551-w

**Published:** 2024-11-07

**Authors:** Chengwang Zhou, Zhezheng Ding, Shengzhe Ying, Hao Jiang, Yan Wang, Timing Fang, You Zhang, Bing Sun, Xiao Tang, Xiaomin Liu

**Affiliations:** 1 School of Textiles and Clothing, School of Chemistry and Chemical Engineering, Qingdao University, Qingdao, 266071 Shandong People’s Republic of China; 2 Centre for Clean Energy Technology, School of Mathematical and Physical Sciences, Faculty of Science, University of Technology Sydney, Ultimo, NSW 2007 Australia; 3 School of Chemistry and Chemical Engineering, Henan Normal University, Xinxiang, 453007 Henan People’s Republic of China

**Keywords:** Zn metal anodes, Double-layered protective film, Electrode/electrolyte optimization, Aqueous zinc-(dual) halogen batteries

## Abstract

Aqueous zinc-halogen batteries are promising candidates for large-scale energy storage due to their abundant resources, intrinsic safety, and high theoretical capacity. Nevertheless, the uncontrollable zinc dendrite growth and spontaneous shuttle effect of active species have prohibited their practical implementation. Herein, a double-layered protective film based on zinc-ethylenediamine tetramethylene phosphonic acid (ZEA) artificial film and ZnF_2_-rich solid electrolyte interphase (SEI) layer has been successfully fabricated on the zinc metal anode via electrode/electrolyte synergistic optimization. The ZEA-based artificial film shows strong affinity for the ZnF_2_-rich SEI layer, therefore effectively suppressing the SEI breakage and facilitating the construction of double-layered protective film on the zinc metal anode. Such double-layered architecture not only modulates Zn^2+^ flux and suppresses the zinc dendrite growth, but also blocks the direct contact between the metal anode and electrolyte, thus mitigating the corrosion from the active species. When employing optimized metal anodes and electrolytes, the as-developed zinc-(dual) halogen batteries present high areal capacity and satisfactory cycling stability. This work provides a new avenue for developing aqueous zinc-(dual) halogen batteries.

**Supplementary Information:**

The online version contains supplementary material available at 10.1007/s40820-024-01551-w.

## Highlights

- A double-layered protective film based on zinc-based coordination compound and ZnF_2_-rich solid electrolyte interphase layer has been successfully fabricated on the zinc metal anode via electrode/electrolyte synergistic optimization.
- The double-layered architecture can effectively modulate Zn^2+^ flux and suppress the zinc dendrite growth, thus facilitating the uniform zinc deposition.
- The as-developed zinc-(dual) halogen batteries based on double-layered protective film can present high areal capacity and satisfactory cycling stability.

**Supplementary Information:**

The online version contains supplementary material available at 10.1007/s40820-024-01551-w.

## Graphical abstract

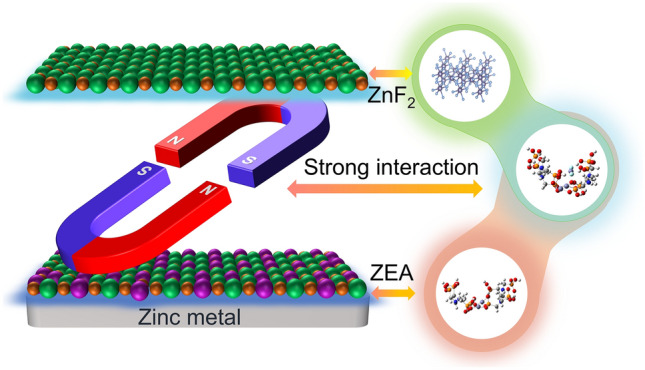

## Introduction

The usage of renewable energies (solar, wind, etc*.*) has attracted tremendous attention with soaring energy demand and environmental pollution [1, 2]. So far, lithium-ion batteries (LIBs) have achieved significant commercial success due to their high energy density [3]. However, the uneven distribution of lithium resources and the highly flammable nature of organic-based electrolytes are obstacles to impede their large-scale deployment [4, 5]. Compared with LIBs, aqueous zinc-based batteries are considered as promising candidates for large-scale energy storage due to the abundant resources, intrinsic safety, and high theoretical capacity of zinc metal anode (5855 mAh cm^−3^ and 820 mAh g^−1^) [6]. Additionally, aqueous electrolytes employed in aqueous zinc-based batteries not only exhibit good compatibility with zinc anode, but also display high ionic conductivities, thereby effectively ensuring the electrochemical performance of aqueous zinc-based batteries [7]. So far, a variety of aqueous zinc-based batteries including zinc-manganese oxide, zinc-vanadium oxide, zinc-Prussian blue analogs, zinc-air, and zinc-halogen batteries have been developed, which are promising development directions for aqueous zinc-based rechargeable batteries in future [8].

Among these aqueous zinc-based batteries, zinc-halogen batteries (*e.g.,*, Zn-I_2_, Zn-Br_2_, and Zn-Cl_2_ batteries) exhibit considerable electrochemical performance based on the halogen-based conversion reactions at the cathode and the plating/stripping of Zn^2+^/Zn at the anode [9]. Typically, the implementation of iodine redox electrochemistry in aqueous zinc-iodine batteries can provide 211 mAh g^−1^ based on the I^−^/I^0^ conversion reaction and 422 mAh g^−1^ based on I^−^/I^0^/I^+^ conversion reaction [10]. Similarly, aqueous Zn-Br_2_ batteries can deliver 335 mAh g^−1^ based on Br^−^/Br^0^ redox pair [11], while a higher theoretical capacity (755 mAh g^−1^) can be achieved by aqueous Zn-Cl_2_ battery based on Cl^−^/Cl^0^ reaction [12]. Particularly, some unique strategies (*e.g.*, construction of zinc-dual-halogen batteries and employment of molten hydrate electrolyte) can further enhance the capacity and energy density of battery configurations [9, 13]. Although aqueous zinc-halogen batteries exhibit superior theoretical capacities and fast reaction kinetics, some inherent drawbacks impedes their further development. Firstly, the relatively narrow electrochemical stability window of traditional aqueous electrolytes may initiate hydrogen evolution reaction (HER), which can change localized pH values and trigger the rapid zinc dendrite growth [14, 15]. Additionally, active species in aqueous zinc-halogen batteries are easily dissolved in aqueous electrolytes during reaction, which can migrate to anode side and corrode the zinc metal [16–18]. This not only continuously consumes active species that leads to rapid capacity attenuation [19], but also accelerates zinc dendrite growth, thus resulting in short lifespan of energy storage devices.

Various strategies, including electrolyte engineering and electrode optimization, have been employed to improve the electrochemical performance of aqueous zinc-halogen batteries [20]. The employment of electrolyte additives to induce the formation of a solid electrolyte interphase (SEI) layer on the surface of the metal anode may enhance the electrochemical performance via isolating the electrode from electrolyte [21]. However, hydrogen evolution during SEI formation may destruct the formed SEI layer, therefore failing to protect the metal anode and leading to reduced Coulombic efficiency [22, 23]. As for electrode optimization, constructing an artificial protective layer can be utilized to block the direct contact between electrode and electrolyte, thus preventing the side reactions and zinc dendrite growth [24]. Nevertheless, the artificial protective layer may suffer from crack and/or degradation due to large volume changes during repeated Zn plating/stripping. Moreover, artificial protective layers are not self-reparable as that of in situ formed SEI, leading to gradually decreased electrochemical performance of Zn anodes [22].

Herein, we demonstrate an in situ structural design of a double-layered protective film on the zinc metal anode via electrode/electrolyte synergistic optimization, which enables high-energy-density and long-cycling aqueous zinc-(dual) halogen batteries. The zinc-ethylenediamine tetramethylene phosphonic acid (denoted as ZEA) coordination compound can be *in situ* synthesized on the zinc metal anode as the inner protective film. Meanwhile, the highly concentrated electrolyte can enlarge the electrochemical stability window, while the tetraethylammonium trifluoromethanesulfonate (TEAOTf) additive could induce the formation of fluorine-rich outer layer. The characterizations reveal that the ZEA-based artificial film possess strong interaction with fluorine-rich SEI layer, which prevents the breakage of SEI film and ensures the structural stability of such architecture. The synergistic cooperation of the coordination compound film and fluorine-rich SEI film not only modulates Zn^2+^ flux and suppresses the zinc dendrite growth, but also the direct contact between metal anode and electrolyte, thus mitigating the corrosion from the active species. When applying optimized metal anode and electrolyte, the as-developed aqueous zinc-iodine batteries can provide an areal capacity of 1.17 mAh cm^−2^ based on four-electron conversion reaction (I^−^/I^0^/I^+^), and maintain 91.1% capacity after 1000 cycles. Furthermore, the aqueous zinc-dual halogen batteries can exhibit a high areal capacity of 2.23 mAh cm^−2^ based on six-electron conversion reactions (I^−^/I^0^/I^+^ and Cl^−^/Cl^0^) and a satisfactory cycling stability (76.5% over 400 cycles).

## Experimental Section

### Materials Preparation

Zinc chloride (ZnCl_2_,99%), iodine (I_2_, ≥ 99.8%), and manganese sulfate (MnSO_4_, analytical reagent) were purchased from Macklin Co., Ltd. Tetraethylammonium triflate (TEAOTf, 98%) was purchased from Energy Chemical, China. Ethylenediamine tetramethylene phosphonic acid (95%) was purchased from Shanghai Aladdin Biochemical Technology Co., Ltd. Carbon cloth (CC) was purchased from Jiaxing Naco New Materials Co., Ltd.

### Preparation of *ZEA*@Zn Metal Anode and Aqueous Electrolytes

Commercial zinc foils with the thickness of 50 μm were repeatedly washed with ethanol and deionized water to remove impurities, which were then dried in the vacuum oven. Subsequently, the pure zinc foil was soaked in 0.1 wt% ethylenediamine tetramethylphosphonic acid (EA) solution for 20 min. Then, ZEA@Zn metal anode can be obtained by rinsing with deionized water for several times. The Cu foil with ZEA layer (ZEA@Cu) can be prepared by soaking Cu foil in 2 wt% ZnCl_2_ + 0.1 wt% EA solution. For preparation of aqueous electrolytes, the 1 m (mol kg^−1^_solvent_) ZnCl_2_ (denoted as E1), 25 m ZnCl_2_ (denoted as E2), and 25 m ZnCl_2_ + 0.005 m MnSO_4_ + 0.1 m TEAOTf (denoted as E3) were prepared by dissolving ZnCl_2_, MnSO_4_, and TEAOTf into deionized water with different formulations, respectively.

### Preparation of I_2_@CC Cathode

The I_2_@CC electrode was prepared by a solution-adsorption method [25]. Briefly, a certain amount of iodine powder was added into the distilled water. A piece of commercial carbon cloth was repeatedly cleaned with anhydrous ethanol and distilled water, which was then soaked in iodine-containing water until the solution becomes clear. The mass loading of iodine on the carbon cloth is around 3 mg cm^−2^.

### Material Characterization

Fourier transform infrared spectroscopy (FT-IR, Nicolet iS50, Thermo Scientific, America) was used to detect the composition and structure of functional groups. X-ray diffraction (XRD, Ultima IV, Rigaku, Japan) using Cu Kα Radiation was used to analyze the composition and structure of materials. The 2θ range was set as 5° ~ 80°, and diffraction data were acquired in step mode of 5°/min. X-ray photoelectron spectroscopy (XPS, ESCALAB Xi + , Thermo Fisher, Czech Republic) with a limit vacuum of 5 × 10^–10^ mbar was performed to detect the valence change and composition of the material. Scanning electron microscopy (SEM, JSM-7800F, JEOL, Japan) and high-resolution transmission electron microscopy (HRTEM, JSM-2100Plus, JEOL, Japan) were used to observe the morphologies of materials. Ultraviolet–visible (UV–vis) spectra were collected by Ultraviolet–visible spectrometer (UV-2600, SHIMADZU, Japan).

### Electrochemical Measurement

Cyclic voltammetry (CV) was used to determine the electrochemical stability window with a three-electrode configuration (Ti mesh as working electrode, Ag/AgCl reference electrode, Pt foil as counter electrode) at a scan rate of 10 mV s^−1^. Linear sweep voltammetry (LSV) was performed with a three-electrode configuration at a scan rate of 0.2 mV s^−1^. EIS measurements were carried out on the CHI 660E electrochemical workstation (Shanghai Chenhua, China) in the frequency range of 10^–2^ ~ 10^5^ Hz. Tafel tests were carried out with Zn or ZEA@Zn as working electrodes at a scan rate of 10 mV s^−1^ within a voltage range of -0.9 ~ 0 V.

The aqueous zinc-iodine batteries and aqueous zinc-(dual) halogen batteries were assembled in the Swagelok cells in the air condition by using the zinc metal anode, I_2_@CC cathode, aqueous electrolytes, and glass fiber as separator (Whatman, GF/F). The electrode areas of the metal anode and I_2_@CC cathode were 0.8 × 0.8 and 0.5 × 0.5 cm^2^, respectively. The CV curves of full cells were tested at a scan rate of 0.3 mV s^−1^ on CHI 660E electrochemical workstation. Cycling performance and rate capability of full cells were performed on a Neware battery test system (CT-4008Tn) at room temperature. Before the electrochemical tests, the as–assembled full cells were first activated by cycling at 6 mA cm^**–**2^ for 10 cycles at room temperature. The voltage ranges of aqueous zinc-iodine batteries and zinc-(dual) halogen batteries were set as 0.6 ~ 1.8 and 0.6 ~ 2.0 V, respectively.

The Zn||Cu asymmetric cells were cycled with current density of 0.5 mA cm^−2^ and plating capacity of 0.5 mAh cm^−2^. The cycle stability of the Zn||Zn symmetric cell was measured by 1 h charging-1 h discharging at a current density of 5 mA cm^−2^, and 0.5 h charging-0.5 h discharging at a current density of 0.5 mA cm^−2^. The electrochemical quartz crystal microbalance (EQCM) measurements were used to monitor the electrode weight change on the CHI 440E electrochemical workstation (Shanghai Chenhua, China). The gold working electrodes were purchased from Shanghai Chenhua, China. Pt wire and saturated sliver/silver chloride (Ag/AgCl) were served as the counter electrode and the reference electrode, respectively. ZEA@Zn on the gold working electrode was prepared by first depositing zinc on the gold working electrode in 1 m ZnCl_2_ electrolyte and then soaking in EA solution for 20 min. All the EQCM tests were conducted at a scan rate of 10 mV s^−1^. Due to the limitations of the high concentration electrolyte and deposition amount, electrolytes were diluted [26, 27]. The mass change (*Δm*) of electrodes was relied on frequency change (*Δf*) according to the Sauerbrey equation [28]:

1 $$\Delta m=-\frac{\text{A}\sqrt{\mu \rho }}{2{f}_{0}^{2}}*\Delta f$$

where ${f}_{0}$ is the resonant frequency of the fundamental mode of the crystal, A is the area of gold disk, μ is the shear modulus of quartz (2.947 × 10^11^ g cm^−1^ s^−2^) and ρ is the density of the crystal (2.648 g cm^−3^). 1 Hz change in frequency corresponds a mass change of 0.0014 μg in this work.

To prepare TEM samples, coin cells were assembled with TEM grid, copper foil, and zinc foil. A film of zinc was first deposited on the TEM grid via electrochemical deposition in 25 m ZnCl_2_ electrolyte; the current density and deposition capacity were 1 mA cm^−2^ and 0.1 mAh cm^−2^, respectively. Then, the TEM grid was disassembled from the coin cell, and was soaked in ethylenediamine tetramethylene phosphonic acid solution to form a ZEA coordination compound layer. Subsequently, the as-obtained TEM grid was assembled into a Zn|E3|Cu asymmetric cell for platting/stripping 1 cycle at current density of 0.5 mA cm^−2^ and deposition capacity of 0.5 mAh cm^−2^. Finally, the TEM sample can be obtained by washing TEM grid with distilled water for several times.

## Results and Discussion

### Design Principle and Structure Characterization

**Fig. 1:**
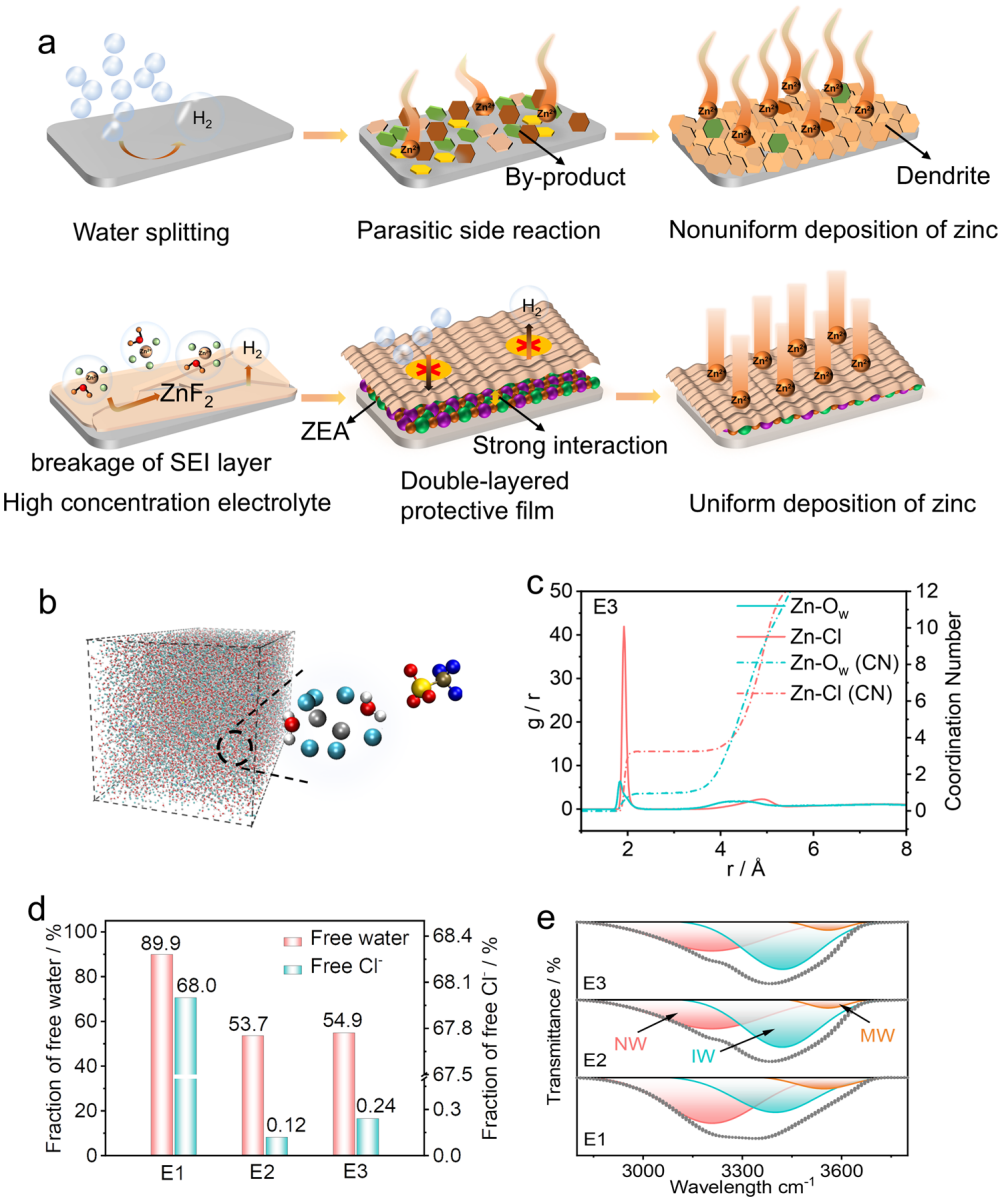
**a** Schematic illustration showing the development of zinc metal anode with double-layered protective film for zinc-(dual) halogen batteries. **b** Snapshots of the MD simulation for the E3 electrolyte. **c** RDF and coordination numbers of Zn–O(H_2_O) and Zn-Cl in the E3 electrolyte. **d** Summary of free water and Cl^−^ content based on MD simulations. **e** FT-IR spectra of different electrolytes

Figure 1a presents the evolution process to optimized zinc metal anodes with the double-layered protective film. Although the zinc metal anode possesses high theoretical capacity and low cost, parasitic side reactions and dendrite growth still hinder its employment. When applying a highly concentrated electrolyte (consisting of 25 m (m: mol kg^−1^_solvent_) ZnCl_2_, 0.005 m MnSO_4_, and 0.1 m TEAOTf) in aqueous zinc-(dual) halogen batteries, the decomposition of TEAOTf additive during the electrochemical process can facilitate the construction of ZnF_2_-rich SEI layer, which exhibiting strong affinity for Zn^2+^ and suppressing the zinc dendrite growth [29]. However, the breakage/reconstruction of such SEI layer in the aqueous electrolyte may lead to continuous consumption of electrolyte and poor cycling performance. Therefore, we further in situ synthesize a ZEA-based artificial film on the Zn anode, which could integrate with ZnF_2_-rich SEI layer to form a double-layered protective film for dendrite-free Zn anodes.

Via in situ synthesizing coordination compound with ethylenediamine tetramethylene phosphonic acid (denoted as EA), a ZEA-based artificial film can be formed on the zinc metal anode, which block the direct contact between electrode and electrolyte, thus preventing the parasitic hydrogen evolution reaction. Furthermore, The ZEA-based artificial film shows strong affinity for the ZnF_2_-rich SEI layer, therefore effectively suppressing the SEI breakage and facilitating the construction of double-layered protective film on the zinc metal anode. Additionally, the in situ formed SEI layer is self-reparable, which in turn protects artificial layer from crack and/or degradation during repeated Zn plating/stripping. Such double-layered architecture can effectively modulate Zn^2+^ flux and suppress the zinc dendrite growth, thus facilitating the uniform zinc deposition [30].

Molecular dynamics (MD) simulation and experimental investigation were carried out to study the solvation structure of aqueous electrolytes (see electrolyte preparation details in experimental section). Figures 1b, c and **S1** present the MD snapshot images and radical distribution function (RDF) curves of 1 m ZnCl_2_ (denoted as E1), 25 m ZnCl_2_ (denoted as E2), and 25 m ZnCl_2_ + 0.005 m MnSO_4_ + 0.1 m TEAOTf (denoted as E3) electrolytes. In 1 m ZnCl_2_ electrolyte, the primary solvation sheath of the individual zinc ion consists of averagely 5.5 water molecules and negligible chloride ion. In the meanwhile, large amounts of water molecules are bonded with each other via hydrogen bonds, and most of chloride ions scatter randomly in the bulk electrolyte. Therefore, free water and free Cl^−^ are as high as ~ 89.9% and 68% in the 1 m ZnCl_2_ electrolyte, respectively (Fig. 1d). In contrast, the RDF curves of concentrated electrolytes (*i.e.,* E2 and E3) disclose that around one water molecules and three chloride ion constitute the zinc solvation sheath, thus significantly decreasing the free water content (~ 53.7% in E2 and ~ 54.9% in E3 electrolytes) and free Cl^−^ content (~ 0.12% in E2 electrolyte and ~ 0.24% in E3 electrolyte). The decreased water content in solvation sheath and bulk electrolyte may effectively improve electrochemical stability of concentrated electrolytes.

Experimental investigations were conducted to study the solvation structure of different electrolytes. Figures 1e and **S2** display the FT-IR spectra of different electrolytes. The broad band located at 3200 ~ 3500 cm^−1^ represents different hydrogen bonding environments [31]. The lowest frequency (~ 3205 cm^−1^) is ascribed to network water (NW) that has H-bond coordination number close to four, while the highest frequency (~ 3567 cm^−1^) can be attributed to multimer water (MW), which is poorly connected to their environment. Additionally, the water molecule that has an average degree of connection can be referred as intermediate water (IW) [32]. When increasing the concentration of the electrolyte, the network water content decreases and the frequency of intermediate water exhibits the blue shift, which indicates the breakage of hydrogen bonds between network water molecules [32]. The electrochemical stability window of the electrolyte was tested by CV (**Fig. S3)**. The high salt concentration in the electrolyte can effectively support the redox reaction in zinc-dual halogen batteries. Furthermore, the ionic conductivity and pH values of E1, E2, and E3 electrolytes are shown in **Fig. S4**. At room temperature, the E3 electrolyte exhibits ~ 21 mS cm^−1^, which is sufficient to support the aqueous zinc-(dual) halogen batteries at current densities of 6 mA cm^−2^.

**Fig. 2:**
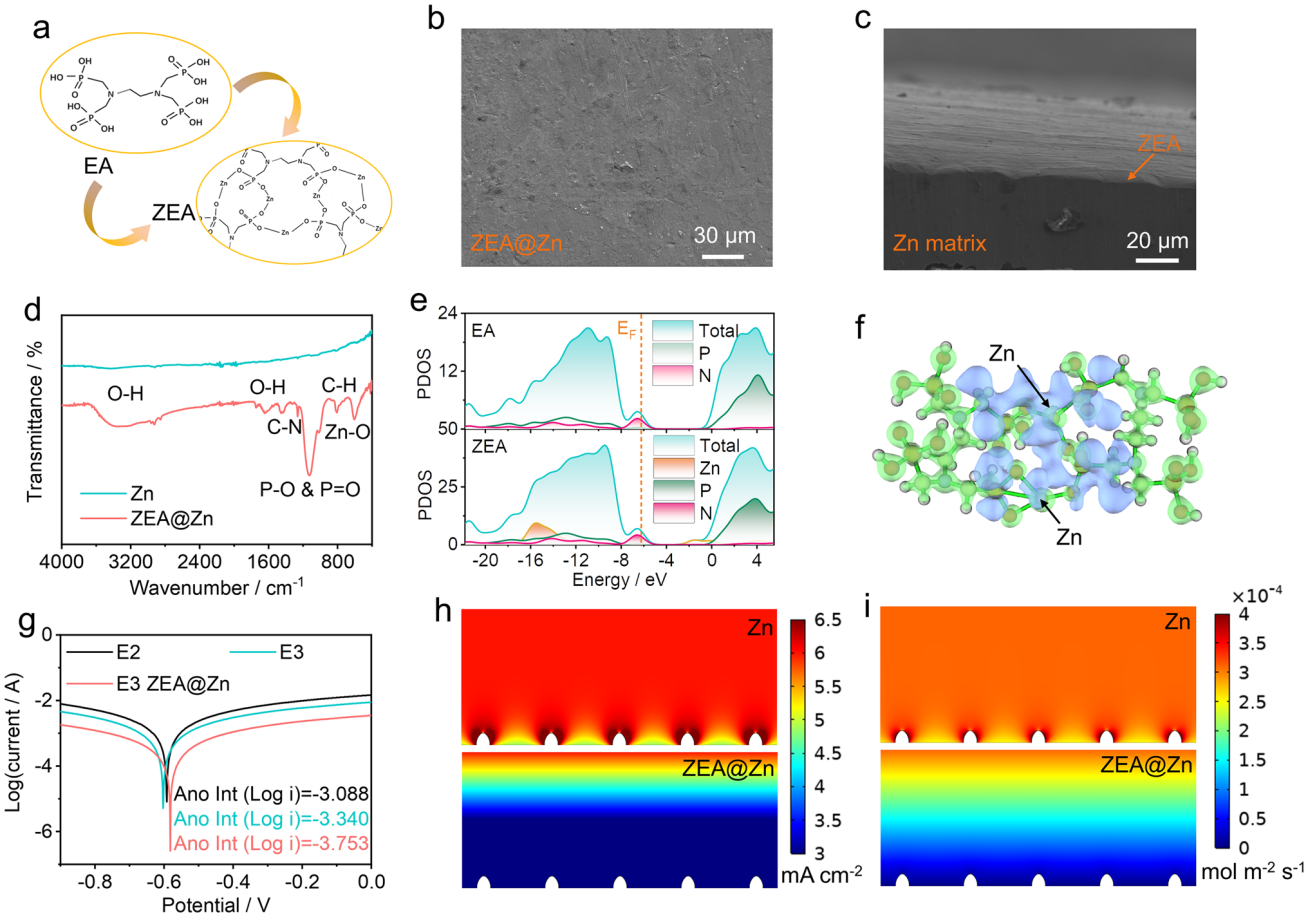
**a** Diagram shows the transformation from ethylenediamine tetramethylene phosphonic acid to zinc-ethylenediamine tetramethylene phosphonic acid. **b, c** Top-view and cross-sectional SEM images of the ZEA@Zn metal anode. **d** FT-IR spectra of pristine Zn and ZEA@Zn metal anodes. **e** DOS of the zinc-based coordination compound. **f** Charge density difference of the zinc-based coordination compound. The light blue represents the electron depletion, while the light green represents the electron accumulation. **g** Corrosion curves of bare Zn and ZEA@Zn anodes in E2 and E3 electrolytes at a scan rate of 10 mV s^−1^. **h** Numerical simulations of current density on bare Zn and ZEA@Zn anodes. **i** Numerical simulations of Zn^2+^ flux distribution on bare Zn and ZEA@Zn anodes

To improve the electrochemical performance of zinc-based batteries, a double-layered protective film based on the ZEA-based artificial film and ZnF_2_-rich SEI layer should be constructed on the zinc metal anode. The schematic illustration for synthesizing artificial film on zinc metal anode is presented in Fig. 2a (see synthesis details in the experimental section). The ethylenediamine tetramethylene phosphonic acid can spontaneously absorb on the zinc metal anode and etch the metal anode, therefore in situ forming a two-dimensional artificial layer on the surface of the Zn anode. Figure 2b, c displays the scanning electron microscopy (SEM) images of the as-treated ZEA@Zn anode. Compared with the pristine Zn anode (**Fig. S5**), a smooth and compact film (ZEA) can be observed on the surface of the Zn anode. The elemental mapping for ZEA@Zn further confirms the successful preparation of artificial film, in which the C, P, O, and N elements are evenly distributed on the surface of the metal anode (**Fig. S6**).

Experimental characterizations, such as FT-IR spectroscopy, XRD, and XPS have been performed to investigate the composition and structure of ZEA@Zn anode. Figure 2d shows the FT-IR spectra of the pristine zinc foil and the ZEA@Zn foil. The ZEA@Zn anode exhibits characteristic peaks at 606, 1120, and 3356 cm^−1^, which corresponds to the Zn–O, P-O/P = O, and O–H bonds, respectively [33–35]. This confirms the successful synthesis of a two-dimensional ZEA-based artificial film on the surface of the zinc metal anode, which is also consistent with the high-resolution XPS spectra (**Fig. S7**). Noticeably, **Fig. S8** shows the XRD patterns of the Zn anode before and after treatment, in which the two samples exhibit similar patterns, and no other peak is observed in ZEA@Zn anode. This suggests the relatively poor crystallinity of artificial film.

The density of states (DOS) for the EA and zinc-based coordination compound (ZEA) have been performed to investigate the electronic conductivity. Both of EA and ZEA are semiconductors with LUMO–HOMO gaps of 4.3 and 2.6 eV, respectively (Fig. 2e). Figure 2f shows the electron density difference of ZEA, in which apparent charge transfer from the EA ligand to zinc ions indicates the strong interaction between metal ions and EA ligand [36]. The anti-corrosion performance of zinc metal anode was also studied by collecting the Tafel curves via a three-electrode configuration. As shown in Fig. 2g, the bare Zn foil exhibits corrosion currents of 0.817 and 0.457 mA in the E2 and E3 electrolytes, respectively. By introducing the artificial layer on the zinc metal anode, the ZEA@Zn anode tested in the E3 electrolyte can achieve a lower corrosion current of 0.177 mA, which means a low corrosion tendency in dynamics [11, 37]. Furthermore, **Fig. S9** shows the LSV measurements on ZEA@Zn and Zn anodes in the E3 electrolyte, in which the smaller reduction current and higher Tafel slope (105.3 mV dec^−1^) for ZEA@Zn indicate the suppressed hydrogen generation owing to the artificial layer.

Such a ZEA-based artificial film is expected to not only block the direct contact between the electrolyte and the electrode, thus suppressing the parasitic side reaction on the electrolyte/electrode interface, but also regulate the Zn^2+^ flux, which leads to uniform nucleation and deposition [38]. To further investigate the inhibition effect of the ZEA-based artificial film on zinc dendrites, the local current density and Zn^2+^ flux distribution within the ZEA layer have been simulated by using COMSOL program (Fig. 2h, i). Initial zinc nuclei were set on the surface of the metal anode with a dimension of 200 nm. The current density of 6 mA cm^−2^ was applied to motivate the Zn^2+^ flux diffusion toward the metal anode. Congestion of zinc ions and uneven distribution of current density can be observed at the neck of the bare zinc metal anode, which accelerates the uneven deposition and continuous dendrite growth (top panels of Fig. 2h, i) [39]. In contrast, the coordination compound layer can effectively homogenize the Zn^2+^ flux distribution and reduces the local current density, therefore inducing the uniform zinc deposition on the metal anode and preventing the dendrite growth (bottom panels of Fig. 2h, i) [11, 40].

### Morphology and Electrochemical Performance of Zinc Metal Anodes

**Fig. 3:**
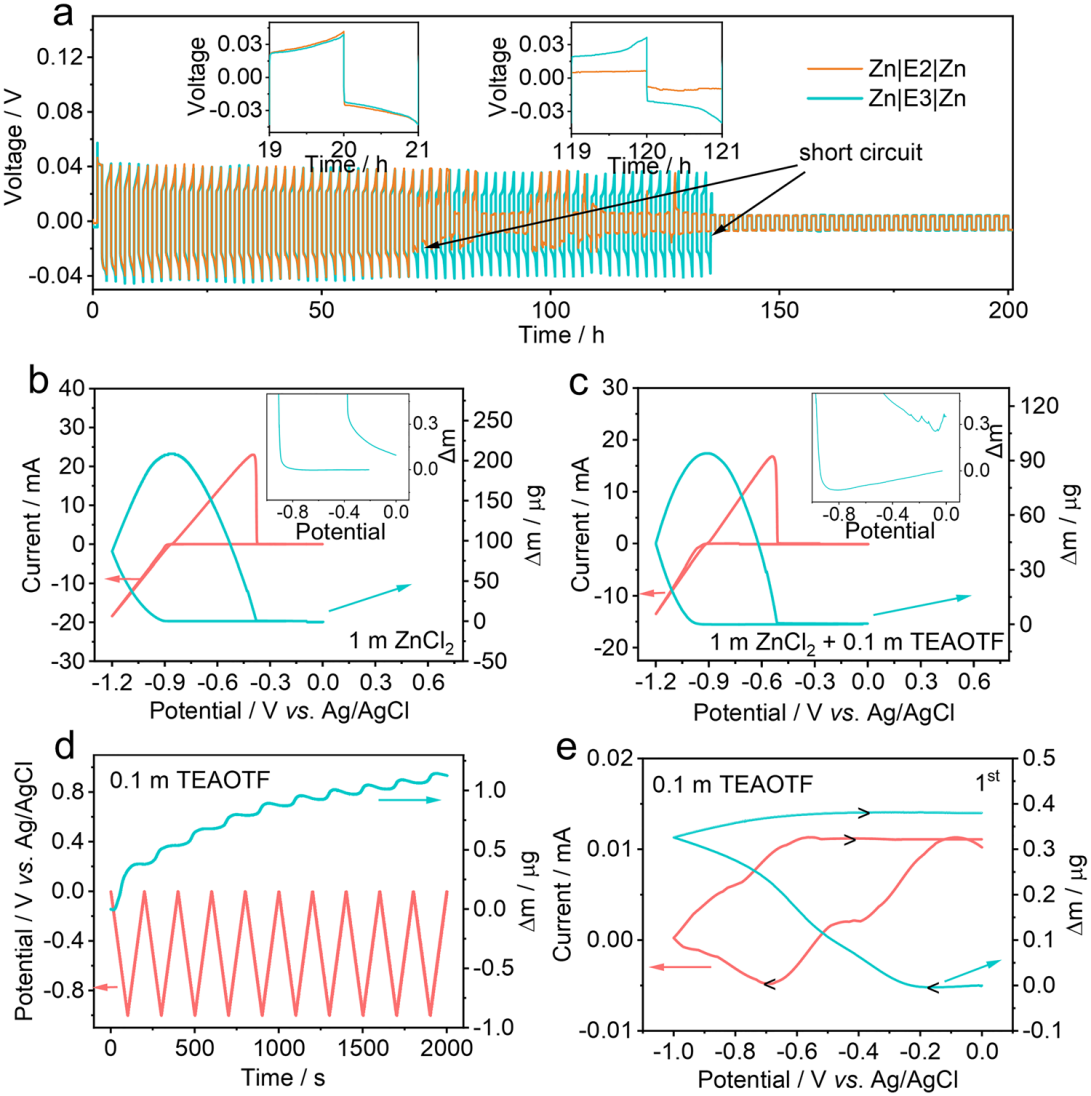
**a** Cycling stability of different symmetric cells (1 h charge-1 h discharge with the current density of 5 mA cm^−2^). The CV curve and the corresponding mass evolution of Au electrode in **b** 1 m ZnCl_2_ and **c** 1 m ZnCl_2_ + 0.1 m TEAOTf electrolytes. **d** Plot for Sauerbrey mass and potential as functions of time during CV measurements at 10 mV s^−1^ in 0.1 m TEAOTf electrolyte. **e** CV curve and the corresponding mass evolution of Au electrode in 0.1 m TEAOTf electrolyte

To verify the electrochemical performance of aqueous electrolytes, symmetric cells were assembled with bare zinc foils and aqueous electrolytes. Figure 3a shows the cycling performance of the symmetric cells, in which the Zn|E2 (25 m ZnCl_2_)|Zn cell experienced a short circuit after cycling for 69 h. By using the E3 (25 m ZnCl_2_ + 0.005 m MnSO_4_ + 0.1 m TEAOTf) electrolyte, a ZnF_2_-rich SEI layer can be formed on the surface of the zinc metal anode due to the decomposition of TEAOTf additive [41]. Such a SEI layer can exhibit strong affinity for Zn^2+^ and suppress the zinc dendrite growth [42, 43]. Therefore, the symmetric cell with the E3 electrolyte exhibits a longer lifespan compared with those with the E2 electrolyte. However, due to the continuous SEI breakage and depletion of additives, the Zn|E3|Zn cell still failed after cycling for 134 h [22].

Electrochemical quartz crystal microbalance (EQCM) measurements were used to monitor the Zn plating/stripping in aqueous electrolytes. Figures 3b, c and **S10** show the electrode mass evolution during CV scanning in 1 m ZnCl_2_ and 1 m ZnCl_2_ + 0.1 m TEAOTf electrolytes. In 1 m ZnCl_2_ electrolyte, 208.6 μg of zinc is platted on the working electrode during the initial cathodic scanning, while the irreversible mass change at the first cycle is 0.1 μg (Fig. 3b). By comparison, the mass evolution curve at the first cycle shows 93.9 μg of zinc plating and 0.35 μg of irreversible mass change when employing 1 m ZnCl_2_ + 0.1 m TEAOTf electrolyte (Fig. 3c), which reflects the relatively lower Coulombic efficiency in 1 m ZnCl_2_ + 0.1 m TEAOTf electrolyte. Such a lower Coulombic efficiency can be ascribed to the decomposition of TEAOTf additive. By performing EQCM tests in 0.1 m TEAOTf electrolyte, the irreversible and continuous weight increase indicates the decomposition of TEAOTf, which leads to fast consumption of additives and relatively poor cycling performance (Fig. 3d, e).

**Fig. 4:**
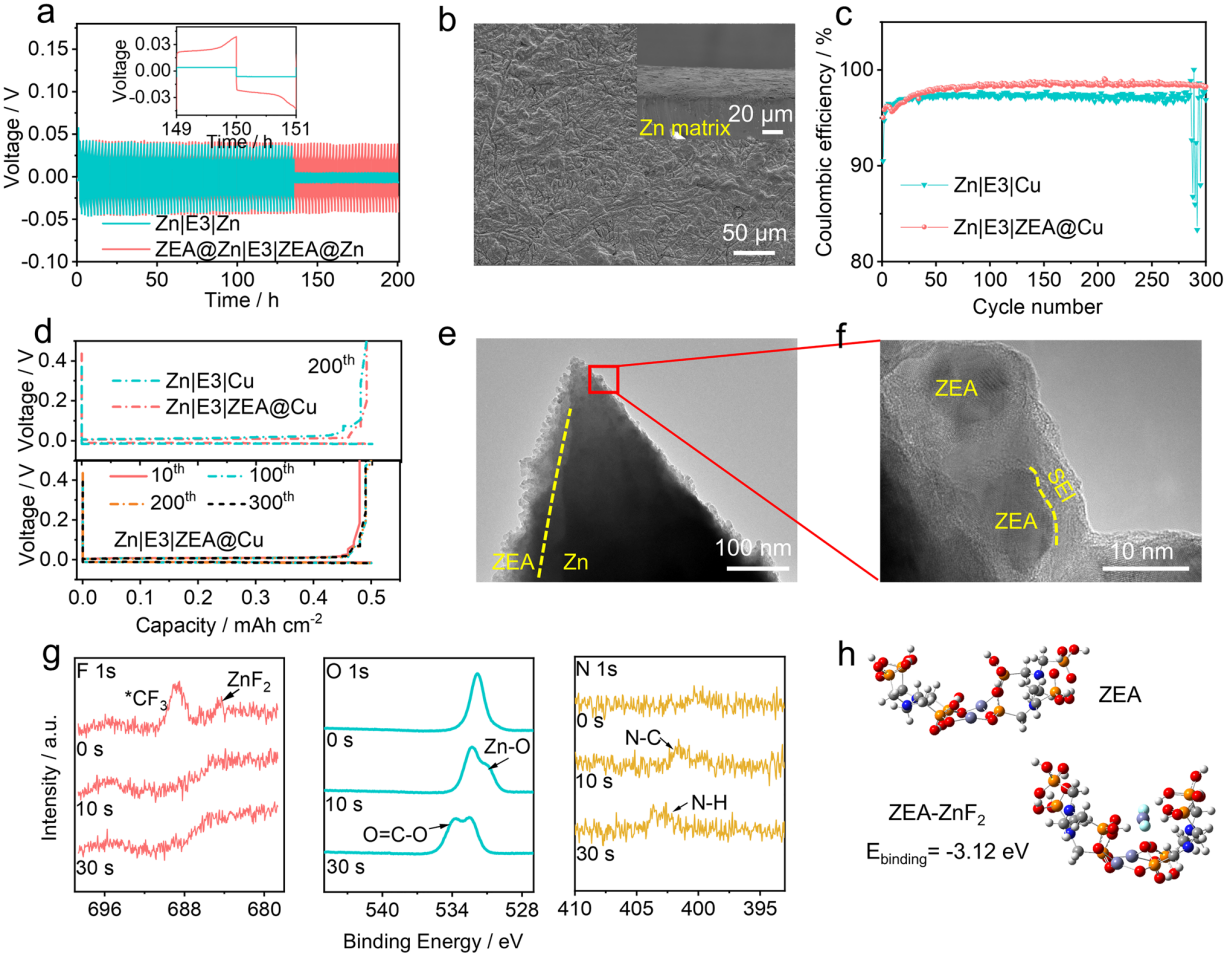
**a** Cycling stability of different symmetric cells (1-h charge–1-h discharge with the current density of 5 mA cm^−2^). **b** Top-view SEM image of ZEA@Zn metal anode after cycling. The inset shows the cross-sectional view of ZEA@Zn metal anode after cycling. **c** Coulombic efficiencies of Zn plating/stripping on bare Cu and ZEA@Cu in the E3 electrolyte with a current density of 0.5 mA cm^−2^ and plating capacity of 0.5 mAh cm^−2^. **d** Voltage profiles of Zn||Cu and Zn||ZEA@Cu cells with the E3 electrolyte (The current density of 0.5 mA cm^−2^ and a deposition capacity of 0.5 mAh cm.^−2^). **e** TEM image of the double-layered protective film on the metal anode. **f** Zoom-in TEM image of the selected area in Fig. 4e. **g** In-depth F 1* s*, O 1* s*, and N 1* s* XPS spectra of the cycled ZEA@Zn anode. **h** Molecular model of ZEA and theoretical calculations showing the interaction between ZEA and ZnF_2_

The benefits of double-layered protective film on the metal anode were further investigated by performing repeated plating/stripping experiments. The symmetric cells were assembled based on ZEA@Zn metal anodes and the E3 electrolyte. As shown in Figs. 4a and **S11**, the Zn|E3|Zn cell short circuits after cycling for 134 h with the current density of 5 mA cm^−2^, while ZEA@Zn|E3|ZEA@Zn cell remains stable over 200 h. Furthermore, the ZEA@Zn|E3|ZEA@Zn cell exhibits a small voltage hysteresis of 28 mV at a current density of 0.5 mA cm^−2^; no obvious hysteresis fluctuation can be found over 500 h (**Fig. S12**). The morphologies of metal anodes disassembled from the cycled symmetric cells can be monitored by SEM (shown in Figs. 4b and **S13**). The Zn anode tested in the E2 electrolyte exhibits uneven surface after cycling. Although the Zn anode tested in the E3 electrolyte exhibit relatively smooth surface, cracks can be formed during cycling. In contrast, a smooth surface without zinc dendrites can be observed on the cycled ZEA@Zn anode. Meanwhile, the formation of a double-layered protective film can also be confirmed by the elemental mapping (**Fig. S14**), in which Zn, O, P, N, F elements are presented on the surface. These results suggest that the formation of a double-layered protective film can effectively regulate the Zn^2+^ flux and suppress the dendrite growth.

Figures 4c, d and **S15** show the Coulombic efficiencies (the ratio of stripping capacity to plating capacity) and the corresponding voltage profiles of the Zn|E2|Cu, Zn|E3|Cu, and Zn|E3|ZEA@Cu asymmetric cells. It is noted that Zn|E2|Cu cell can achieve a Coulombic efficiency of ~ 98.1%, while the bare Cu foil exhibits a relatively lower Coulombic efficiency (~ 97%) in the E3 electrolyte, which strongly suggests the formation of SEI layer due to the TEAOTf decomposition. Noticeably, the Coulombic efficiency of the Zn|E3|Cu cell drops dramatically after 270 cycles, which can be attributed to the continuous breakdown/reconstruction of SEI layers and dendrite growth [44]. In contrast, the Cu foil with the double-layered protective film (ZEA@Cu; see preparation details in experimental section) can achieve an improved Coulombic efficiency (~ 98.5%) and cycling stability, which is higher than those of Zn|E3|Cu cells. This suggests that the strong chemical interaction between coordination compound and ZnF_2_ can suppress the SEI dissolution in the aqueous electrolytes. Noticeably, the Coulombic efficiency of 98.5% indicates that side reactions still occur during stripping and deposition. The presence of small amount of [Zn(OH_2_)_6_]^2+^ complexes in the electrolyte may cause the formation of electrochemical non-active Zn(OH)_2_ and ZnO, thus decreasing the Coulombic efficiency [45, 46]. Additionally, although the strong chemical interaction in double-layered architecture can suppress the SEI dissolution into the aqueous electrolytes to some extent, small amount of SEI breakage/reconstruction may also deteriorate the Coulombic efficiency.

The visual observation of the double-layered structure can be realized by TEM measurements. The schematic illustration is presented in **Fig. S16**. Briefly, a film of zinc was first deposited on the copper TEM via electrochemical deposition, which was then transferred to ethylenediamine tetramethylene phosphonic acid solution to form a ZEA-based artificial film. After stripping/stripping for 1 cycle in the E3 electrolyte, the TEM grid was disassembled from the cell for TEM observation (see preparation details in the experimental section). As shown in Fig. 4e, the ZEA-based artificial film can be observed to form on the surface of the zinc metal anode. Furthermore, Fig. 4f shows zoom-in TEM image of the selected area in Fig. 4e. A ZnF_2_-rich layer with a thickness of ~ 5 nm can be detected on the surface of ZEA coordination compound, which is related to the decomposition of OTf^−^ anions. This is also confirmed by the FT-IR (**Fig. S17**) and in-depth XPS spectra (Fig. 4g). In F 1*s* XPS spectra, two peaks at 688.9 and 684.5 eV can be ascribed to the -CF_3_ and ZnF_2_, respectively [47]. Noticeably, the F 1*s* signals disappear after etching process, which demonstrates that the ZnF_2_-rich SEI film mainly distributes at the outer layer. Additionally, Zn–O bond (at 531 eV) in the O 1*s* spectra and C-N (at 401.2 eV) in the N 1*s* spectra emerge after etching the Zn anode for 10 s, which indicates the existence of ZEA at the inner layer [48, 49]. Noticeably, the O = C-O signal at 534 eV in the O 1*s* can be ascribed to the Zn_5_(CO_3_)_2_(OH)_6_ on the surface of metal anode, which is reflected in the C 1*s* XPS spectra (**Fig. S18**) [22].

The interaction between the ZEA-based artificial film and ZnF_2_-rich SEI layer can be further studied by EQCM measurements. **Figure S19** shows a mass evolution curve of the ZEA@Zn in 1 m ZnCl_2_ + 0.1 m TEAOTf electrolyte, in which 45.36 μg of mass increase during cathodic scanning and 0.1 μg of irreversible mass change at the first cycle are detected. Such good reversibility strongly suggests that the double-layered protective film based on coordination compound and ZnF_2_-rich SEI layer can effectively suppress the dendrite growth and prolong the cycle life of the metal anode. Noticeably, a small electrode mass decrease can be observed at ~ 1 V, which can be ascribed to expel of anions (Cl^−^ and OTf^−^) absorbed in the ZEA coordination compound layer. Density functional theoretical (DFT) shown in Fig. 4h reveals the strong chemical interaction between coordination compound and ZnF_2_ (-3.12 eV), implying the stability of the double-layered protective film. This could suppress the dissolution of ZnF_2_ in the aqueous electrolytes, thus effectively ensuring the structural stability of the double-layered protective film.

### Electrochemical Performance of Aqueous Zinc-(Dual) Halogen Batteries

**Fig. 5:**
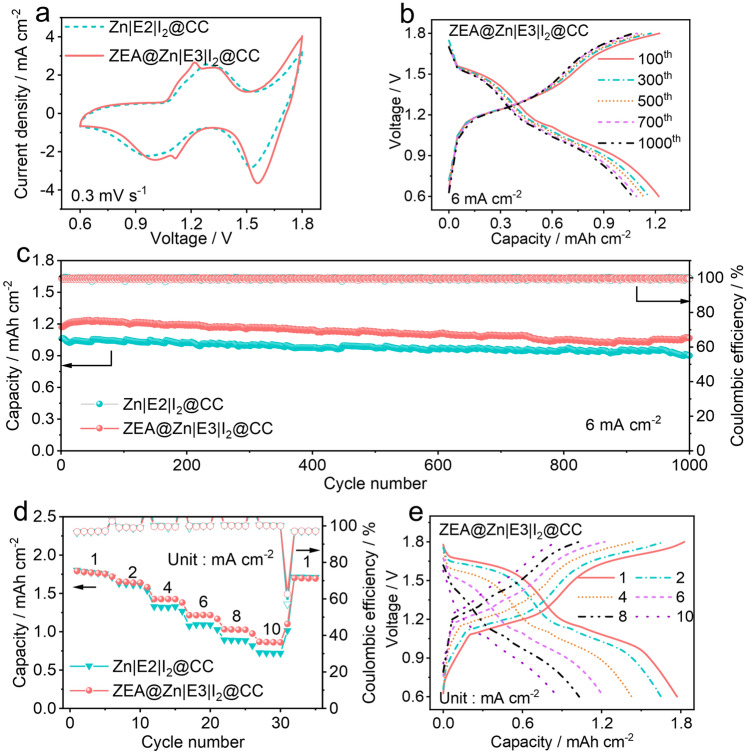
Electrochemical performance of the four-electron aqueous zinc-iodine batteries with a working voltage of 0.6 ~ 1.8 V. **a** CV curves of Zn|E2|I_2_@CC and ZEA@Zn|E3|I_2_@CC cells at a scanning rate of 0.3 mV s^−1^. **b** Voltage profiles of a ZEA@Zn|E3|I_2_@CC cell at different cycles. **c** Cycling performance of Zn|E2|I_2_@CC and ZEA@Zn|E3|I_2_@CC cells at a current density of 6 mA cm^−2^. **d** Rate performance of Zn|E2|I_2_@CC and ZEA@Zn|E3|I_2_@CC cells at different current densities. **e** The corresponding voltage profiles of a ZEA@Zn|E3|I_2_@CC cell at different current densities

To verify the electrochemical performance of the double-layered protective film, four-electron aqueous zinc-iodine batteries have been developed by using zinc metal anodes, I_2_@CC cathode, and aqueous electrolytes. When the cutoff voltage set up as 0.6 ~ 1.8 V, a four-electron conversion reaction (*i.e.*, I^−^/I^0^/I^+^) could occurs in the aqueous zinc-halogen batteries. As shown in Figs. 5a and **S20**, CV curves of aqueous zinc-iodine batteries present two pairs of redox peaks at 1.33/1.01 V and 1.78/1.56 V, which can be attributed to the I^−^/I^0^, I^0^/I^+^ conversion reactions, respectively [10, 50]. Noticeably, in ZEA@Zn|E3|I_2_@CC cells, a pair of small peaks located at 1.22/1.11 V can be observed, corresponding to the insertion of zinc ions into the interlayer of MnO_2_ that derived from the oxidation of MnSO_4_ additive during initial charging process (see detailed discussion in **Fig. S21**) [51]. The MnO_2_ formed during charging process can act as an adsorbent to restrain active species, therefore improving the electrochemical performance of batteries [52, 53].

Figures 5b, c and **S22** show the cycling performance and the corresponding voltage profiles of aqueous zinc-iodine batteries. The aqueous zinc-iodine batteries with bare Zn foil and E2 electrolyte (25 m ZnCl_2_) can deliver a capacity of 1.06 mAh cm^−2^ (353 mAh g^−1^ based on the mass of iodine), and maintain 84.8% after 1000 cycles. By using the E3 electrolyte, the Zn|E3|I_2_@CC cell exhibits an improved capacity (1.17 mAh cm^−2^, 390 mAh g^−1^ based on the mass of iodine). This indicates that the ZnF_2_-rich SEI layer derived from additive decomposition can improve the performance of the battery by suppressing dendritic growth. Meanwhile, the MnO_2_ adsorbent derived from MnSO_4_ additive can restrain the shuttling of active species, thus improving the battery capacity [53]. However, relatively poorer cycling performance (77.9% capacity retention after 1000 cycles) can be observed in the Zn|E3|I_2_@CC cell, which may be ascribed to the continuous SEI breakage and consumption of TEAOTf additives.

By adopting ZEA@Zn metal anode and the E3 electrolyte, the aqueous zinc-iodine batteries exhibit a capacity of 1.17 mAh cm^−2^ (390 mAh g^−1^ based on the mass of iodine). Moreover, an improved capacity retention of 91.1% can be achieved after 1000 cycles, which is better than those of Zn|E2|I_2_@CC and Zn|E3|I_2_@CC cells (shown in Figs. 5c and **S22**). This indicates that the double-layered protective film induced by the electrode/electrolyte co-optimization can effectively facilitate the enhancement of the electrochemical performance. Additionally, the rate performance of the as-assembled batteries was evaluated (shown in Figs. 5d, e and **S23**). With the double-layered structure on the surface of the metal anode, the batteries present superior rate capability. Considerable capacities of 1.77, 1.65, 1.43, 1.21, and 1.03 mAh cm^−2^ can be achieved at the current densities of 1, 2, 4, 6, and 8 mA cm^−2^, respectively. Even at a high current density of 10 mA cm^−2^, the aqueous zinc-iodine batteries can deliver 0.86 mAh cm^−2^, which is better than its counterparts (Zn|E2|I_2_@CC and Zn|E3|I_2_@CC cells).

**Fig. 6:**
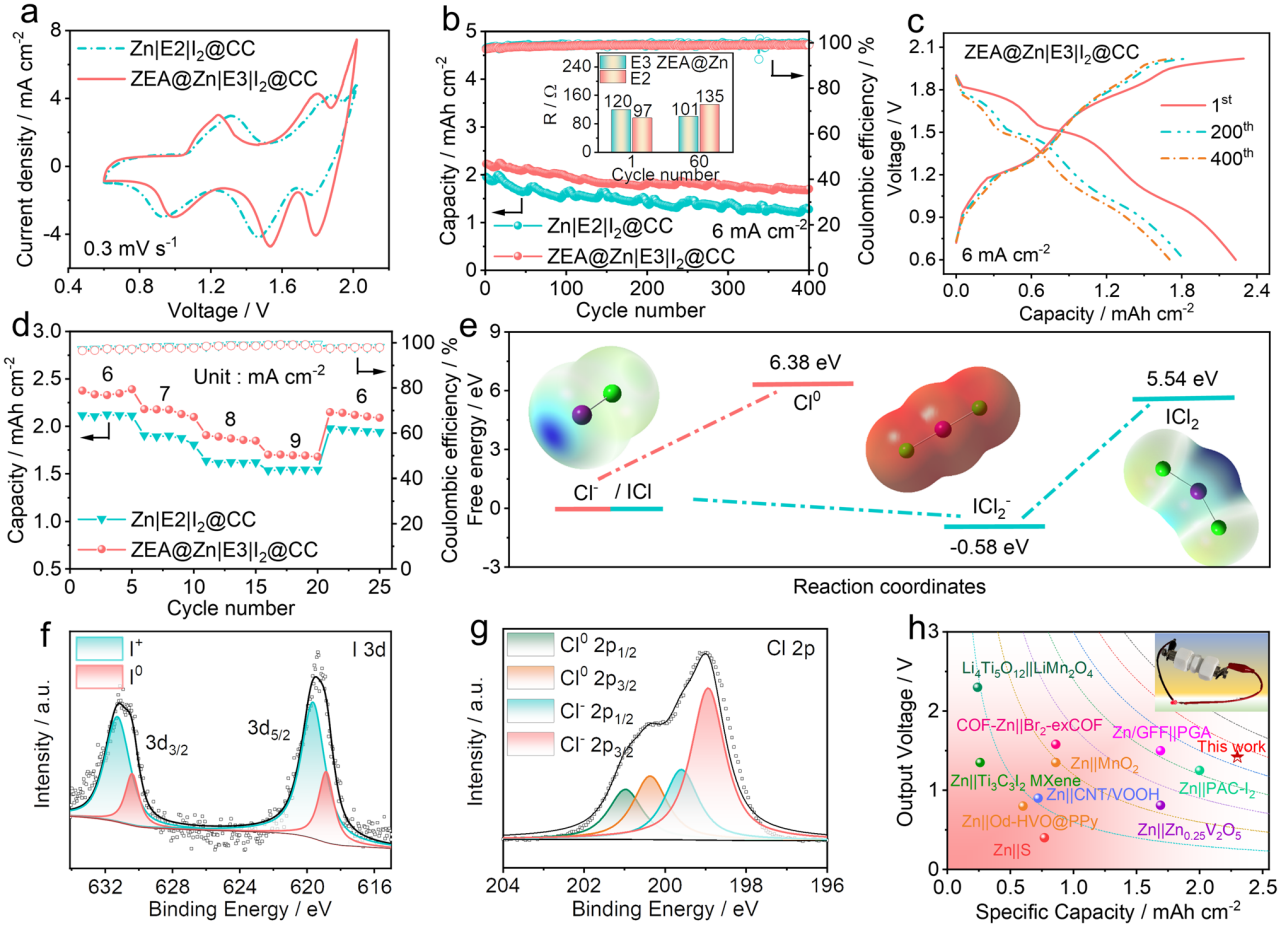
Electrochemical performance of the aqueous zinc-dual halogen batteries with a working voltage of 0.6 ~ 2.0 V. **a** CV curves of the aqueous zinc-dual halogen batteries at a scanning rate of 0.3 mV s^−1^. **b** Cycling performance of zinc-dual halogen batteries at a current density of 6 mA cm^−2^. The inset shows the EIS results after cycling. **c** The corresponding voltage profiles of the ZEA@Zn|E3|I_2_@CC cell at different cycles. **d** Rate performance of different aqueous zinc-dual halogen batteries. **e** Possible reaction paths for Cl^−^ in the bulk electrolyte. High-resolution **f** I 3*d* and **g** Cl 2*p* XPS spectra of a I_2_@CC cathode at fully charged state. **h** Comparison of electrochemical performance of various rechargeable batteries

When tuning the cutoff voltage to 0.6 ~ 2.0 V, six-electron conversion reactions (*i.e.*, I^−^/I^0^, I^0^/I^+^, and Cl^−^/Cl^0^) can occur successively, therefore realizing the dual–halogen chemistry in the batteries. Figures 6a and **S24** show CV curves of aqueous zinc-dual halogen batteries at a scanning rate of 0.3 mV s^−1^. Three redox pairs can be observed to be located at ~ 1.25/0.99, 1.80/1.55, and 1.99/1.79 V, which can be attributed to the I^−^/I^0^, I^0^/I^+^, and Cl^−^/Cl^0^, respectively. Noticeably, the aqueous zinc-dual halogen battery with the ZEA@Zn metal anode and the E3 electrolyte exhibits increased current density, indicating the improved energy density of the battery system. The cycling performance of aqueous zinc-dual halogen batteries was evaluated by repeated charging/discharging at a current density of 6 mA cm^−2^ (shown in Figs. 6b, c and **S25**. Zn|E2|I_2_@CC cells can exhibit an areal capacity of 1.96 mAh cm^−2^ (653 mAh g^−1^ based on the mass of iodine) and capacity retention of 65.3% after 400 cycles. By adopting the E3 electrolyte, the aqueous zinc-dual halogen battery can deliver a higher capacity of 2.48 mAh cm^−2^ (827 mAh g^−1^ based on the mass of iodine), which may be ascribed to the formation of ZnF_2_-rich SEI layer on the zinc metal anodes and MnO_2_ adsorbent on the cathode. Noticeably, the UV–vis spectra (shown in **Fig. S26**) shows that chlorinated by-products in the E3 electrolyte are significantly decreased, which indicates the adsorption of MnO_2_ toward active species.

It is noted that the ZEA@Zn|E3|I_2_@CC cells exhibit a relatively lower capacity (2.23 mAh cm^−2^, 744 mAh g^−1^ based on the mass of iodine) compared with Zn|E3|I_2_@CC cells, which is because the artificial protective film on the metal anode slightly increases the interphase resistance. This is confirmed by the symmetric cells (Figs. 4a and **S11**). However, the continuous dissolution of inorganic SEI layer in Zn|E3|I_2_@CC cells leads to a poor cycling performance (62.7% after 300 cycles); While the double-layered protective film in the ZEA@Zn|E3|I_2_@CC cells can display an improved cycling performance (76.5% capacity retention after 400 cycles) owing to the interaction between ZEA and inorganic SEI. Noticeably, even after 300 cycles, Cl^−^/Cl^0^ related discharge plateau can still be preserved with a capacity of 0.38 mAh cm^−2^. This implies that the double-layered structure induced by electrode/electrolyte co-optimization not only effectively suppresses the dendrite growth, but also mitigates the parasitic side reactions during the charging/discharging process, therefore benefiting the long-term cycling stability of the aqueous zinc-dual halogen batteries. The effect of etching time on the zinc metal anode is also investigated by extending the etching time. Noticeably, the extension of etching time may significantly increase the battery polarization, therefore deteriorating the capacity (**Fig. S27**).

Furthermore, electrochemical impedance spectroscopy (EIS) was measured to investigate the interfacial properties of batteries. The inset of Figs. 6b and **S28** shows the EIS results of zinc-dual halogen batteries after different cycles. The ZEA@Zn|E3|I_2_@CC cell shows reduced resistance after cycling, which suggests that the shuttle effect of active species can be effectively inhibited by the double-layered protective film. Figures 6d and **S29** show the rate performance and the corresponding voltage profiles of aqueous zinc-dual halogen batteries. Compared with the Zn|E2|I_2_@CC cell, the Zn|E3|I_2_@CC cell can deliver improved capacities of 2.56, 2.08, 1.99, and 1.82 mAh cm^−2^ at current densities of 6, 7, 8, and 9 mA cm^–2^, respectively, which confirms better reaction kinetics due to the electrolyte additives. Additionally, the employment of the ZEA@Zn metal anode in aqueous zinc-dual halogen batteries can still maintain such enhanced rate capability.

The underlying reaction mechanism on the cathode was examined by DFT calculations and XPS spectra. Figure 6e presents the possible reaction paths of Cl^−^ oxidation by calculating the potential molecular configurations and corresponding free energies. One of the possible paths is the oxidation of single Cl^−^ to Cl^0^, while the free energy difference of this reaction is 6.38 eV. By comparison, when I^+^ ions are generated in the aqueous zinc-dual halogen batteries, interhalogens can be formed by bonding I^+^ with Cl^−^. Noticeably, the free energy difference between [ICl_2_]^−^ and ICl is -0.58 eV, which implies the spontaneous conversion from ICl to [ICl_2_]^−^. Therefore, [ICl_2_]^−^ interhalogen is thermodynamic stable as an intermediate product, which is similar to previously reported work [54, 55]. Additionally, the free energy difference of the interhalogen oxidation (from [ICl_2_]^−^ to [ICl_2_]^0^) is 6.12 eV, which is lower than that of Cl^−^/Cl^0^ (6.38 eV). As a result, the presence of I^+^ ions can facilitate the oxidation of Cl^−^ following the blue reaction path, which explains why no Cl_2_ gas is produced during the charge process. The XPS measurements were carried out to characterize the I_2_@CC cathode at a fully charged state in the E3 electrolyte. As shown in Fig. 6f, I *3d* XPS spectrum shows I 3*d*_5/2_ doublet at 619.3 eV and I 3*d*_3/2_ doublet at 630.3 eV, both of which can be deconvolved to I^0^ and I^+^ signals. This confirms the I^−^/I^0^/I^+^ conversion reaction at the lower-ordered plateaus [56]. Additionally, Fig. 6g shows the Cl 2*p* XPS spectrum, in which the peaks at 201, 200.3, 199.6, and 198.9 eV are ascribed to Cl^0^ 2*p*_1/2_, Cl^0^ 2*p*_3/2_, Cl^−^ 2*p*_1/2_, and Cl^−^ 2*p*_3/2_, indicating the interhalogen oxidation at the higher-ordered plateau [56]. This can also be verified by ex situ Raman spectra (shown in **Fig. S30)**. Figure 6h and Table S1 compare the electrochemical performance of the as-developed zinc-dual halogen battery with previously reported energy storage devices. The zinc-dual halogen battery presents a high areal capacity and comparable output voltage. Owing to its intrinsic safety and low cost, the aqueous zinc-dual halogen battery is a promising candidate for the large-scale energy storage.

## Conclusion

In summary, a double-layered protective film has been successfully fabricated on the zinc metal anode via electrode/electrolyte synergistic optimization. The in situ synthesized ZEA-based artificial film shows strong affinity for ZnF_2_-rich SEI layer, therefore effectively suppressing the SEI breakage and facilitating the construction of double-layered protective film on the zinc metal anode. Such double-layered protective film based on coordination compound film and inorganic-rich SEI layer not only regulates uniform Zn^2+^ flux to suppress the growth of zinc dendrite, but also significantly restrains the parasitic side reactions by blocking the direct contact between metal anode and active species. Therefore, the as-developed zinc-(dual) halogen batteries can provide a high areal capacity of 1.17 mAh cm^−2^ based on four-electron conversion reaction (I^−^/I^0^/I^+^) and 2.23 mAh cm^−2^ based on six-electron conversion reactions (I^−^/I^0^/I^+^ and Cl^−^/Cl^0^). Furthermore, the as-assembled zinc-dual halogen battery can achieve satisfactory cycling stability (76.5% over 400 cycles) and energy density (3.16 mWh cm^−2^), which endows this aqueous zinc-based battery as a promising candidate for the large-scale energy storage.

## Supplementary Information

Below is the link to the electronic supplementary material.

Supplementary file1 (DOCX 14529 KB)
