# Supplementary material for: Electrode/Electrolyte Optimization-Induced Double-Layered Architecture for High-Performance Aqueous Zinc-(Dual) Halogen Batteries
Source: Nanomicro Lett. 2024 Nov 7;17:58. doi: 10.1007/s40820-024-01551-w (PMC11544112; doi:10.1007/s40820-024-01551-w)
Supplement: Supplementary file 1 — Supplementary file1 (DOCX 14529 KB) [file 40820_2024_1551_MOESM1_ESM.docx]

**S**upporting Information for

**Electrode/Electrolyte Optimization Induced-Double Layered Architecture for High-Performance Aqueous Zinc-(Dual) Halogen Batteries**

Chengwang Zhou^1,#^, Zhezheng Ding^1,#^, Shengzhe Ying,^a^ Hao Jiang^1^, Yan Wang^1^, Timing Fang^1^, You Zhang^1^, Bing Sun^2^, Xiao Tang^1,^* and Xiaomin Liu^1,3,^ *

^1^School of Textiles and Clothing, School of Chemistry and Chemical Engineering, Qingdao University, Qingdao 266071, Shandong, P. R. China

^2^Centre for Clean Energy Technology, School of Mathematical and Physical Sciences, Faculty of Science, University of Technology Sydney, Ultimo, NSW 2007, Australia

^3^School of Chemistry and Chemical Engineering, Henan Normal University, Xinxiang 453007, Henan, P. R. China

^#^Chengwang Zhou and Zhezheng Ding contributed equally to this work.

*Corresponding authors. E-mail: tangxiao@qdu.edu.cn (Xiao Tang); liuxiaomin@qdu.edu.cn (Xiaomin Liu)

**S1 Computational Details**

Molecular dynamics (MD) simulations were conducted to study the solvation structures of different electrolytes [S1]. The MD simulations were run by using LAMMPS. The systems are setup initially by using PACKMOL and Moltemplate (<http://www.moltemplate.org/>) [S2]. The properties of H_2_O are assessed with SPC/E parameters. The force-fields parameters and partial charges of Zn^2+^ and Cl^-^ are taken from previous publications [S3-S6]. The compositions of simulated electrolytes are given in **Table S2**.

The DFT-D3 calculation was carried out using the Gaussian 09 software [S7].The structural optimization was performed at M06-2X/def2-TZVP level, which has been proved to be reliable in identifying molecular interactions [S8]. Vib rational frequencies were calculated at the same level of theory to ensure that the optimized structures have no imaginary frequency as the global minima. The Gibbs free energy changes (ΔG) were calculated by:

ΔG = G_P_ - G_R_  (S1)

where G_R_ is the total Gibbs free energy of reactants and G_P_ is the total Gibbs free energy of the products. The Gibbs free energies were the correction of single-point energies, which were calculated at M06-2X/def2-TZVP level. The continuum solvation model SMD was used to simulate the reaction environment in an aqueous phase, where the solvent was set as water [S9].

The transfer behavior of Zn^2+^ inside the ZEA layer based on the Nernst-Planck equation was simulated using the tertiary current distribution interface of COMSOL Multiphysics 5.5 (the governing equations are shown in Eqs. S2-S6). The transport and reaction behaviors of zinc ions within the ZEA layer were calculated based on the Butler-Volmer kinetic equation. A two-dimensional model includes a 23 μm-thick electrolyte domain, a 2 μm-thick ZEA layer, and several 200 nm-high bump on the electrode surface. The initial concentration of the electrolyte was 25 M and the current density at the cathode on the upper surface was set as 6 mA/cm^2^.

$\text{∇}\text{∙}\text{J}_{\text{i}}\text{+}\text{u}\text{∙}\text{∇}\text{c}_{\text{i}}\text{=}\text{R}_{\text{i}}$ (S2)

$\text{∇}\text{∙}\text{i}_{\text{l}}\text{=}\text{ε}_{\text{l}}\text{F}\sum_{\text{i}} \text{z}_{\text{i}}\text{R}_{\text{i}}\text{+}\text{Q}_{\text{l}}$ (S3)

$\text{∇}\text{∙}\text{i}_{\text{s}}\text{=-}\text{ε}_{\text{l}}\text{F}\sum_{\text{i}} \text{z}_{\text{i}}\text{R}_{\text{i}}\text{+}\text{Q}_{\text{s}}$ (S4)

$\sum_{\text{i}} \text{z}_{\text{i}}\text{c}_{\text{i}}\text{=0}$ (S5)

$\text{J}_{\text{i}}\text{=}\text{-D}_{\text{i,eff}}\text{∇}\text{c}_{\text{i}}\text{-}\text{z}_{\text{i}}\text{u}_{\text{m,j,eff}}\text{F}\text{c}_{\text{i}}\text{∇}\text{ϕ}_{\text{l}}$ (S6)

where $\text{J}_{\text{i}}$ is i ion flux, **u** is flow rate, $\text{c}_{\text{i}}$ is i ion concentration, $\text{R}_{\text{i}}$ is the reaction term, $\text{i}_{\text{l}}\text{=F}\sum_{\text{i}} {\text{z}_{\text{i}}\text{J}}_{\text{i}}$ is electrolyte current density, F is Faraday′s constant, $\text{z}_{\text{i}}$ is the number of charges, $\text{Q}_{\text{l}}$ is the electrolyte current source term, $\text{i}_{\text{s}}\text{=-}\text{σ}_{\text{s,eff}}\text{∇}\text{φ}_{\text{s}}$ is electrode current density, $\text{Q}_{\text{s}}$ is the electrode current source term, $\text{D}_{\text{i,eff}}$ is ion diffusion coefficient, $\text{u}_{\text{m,j,eff}}$ is ion mobility, and $\text{ϕ}_{\text{l}}$ is electrolyte potential.

**S2 Supplementary Figures and Tables**


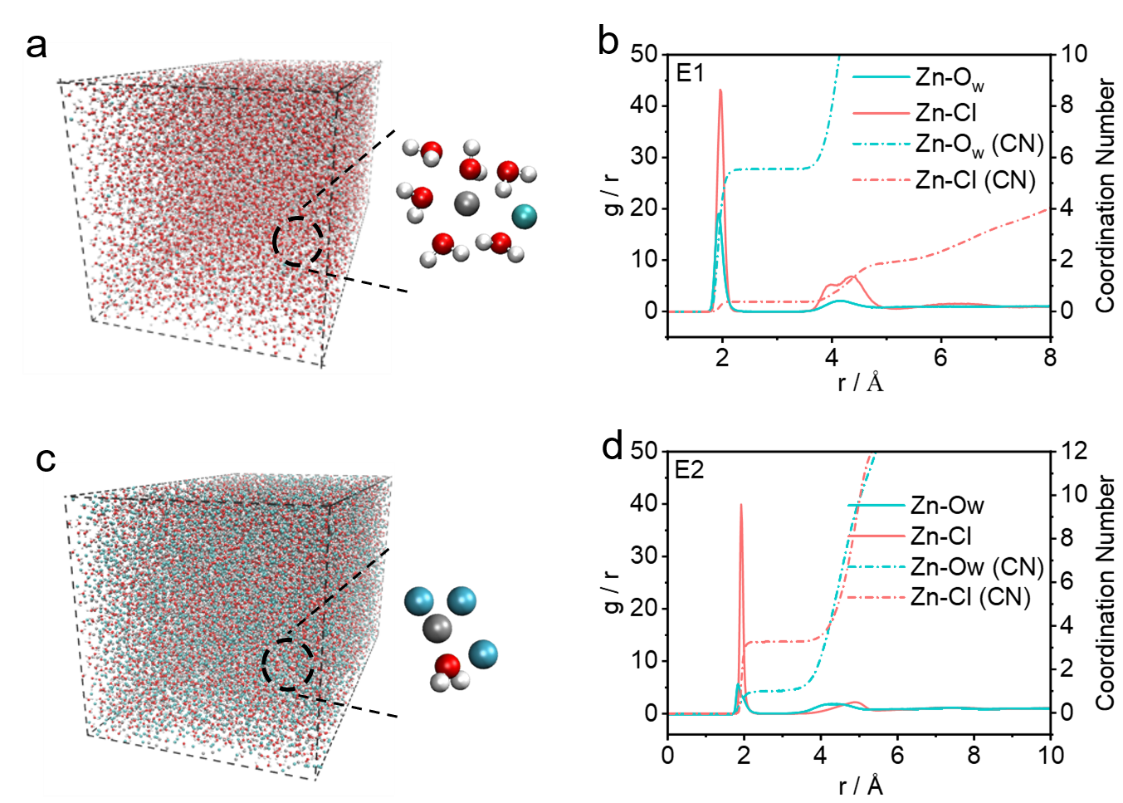


**Fig. S1** (**a**) Snapshot image of the E1 electrolyte obtained from the MD simulation. (**b**) The RDF and coordination numbers of Zn-O(H_2_O) and Zn-Cl in the E1 electrolyte. (**c**) Snapshot image of the E2 electrolyte obtained from the MD simulation. (**d**) The RDF and coordination numbers of Zn-O(H_2_O) and Zn-Cl in the E2 electrolyte


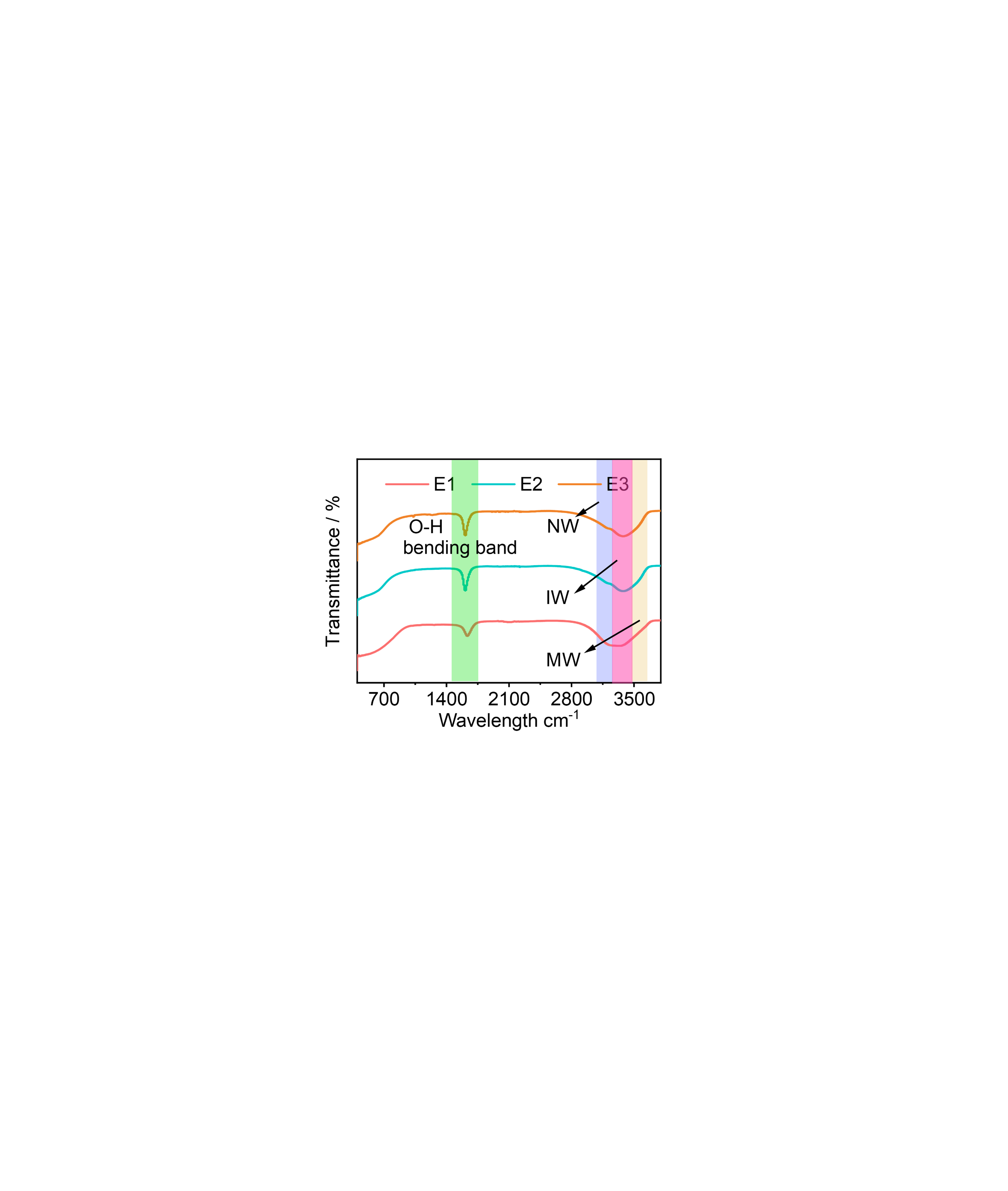


**Fig. S2** FTIR spectra of different aqueous electrolytes. The characteristic peak at 1610 cm^-1^ can be assigned to the O-H bending band, while the broad band ranging from 3100~3600 cm^-1^ corresponds to the O-H stretching band. Noticeably, the broad band at 3100~3600 cm^-1^ is composed of three different hydrogen bonding environments, which includes network water, intermediate water, and multimer water


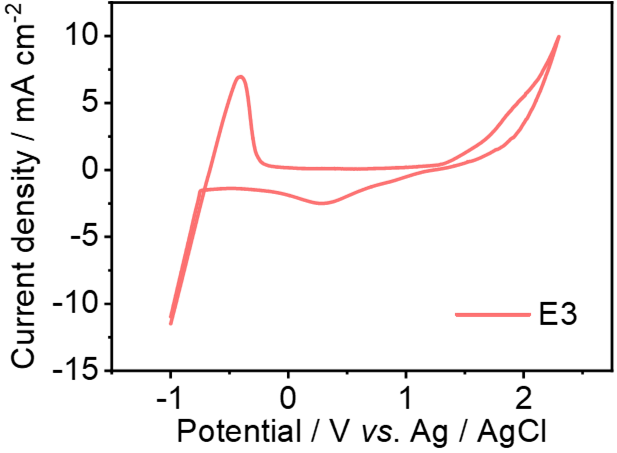


**Fig. S3** CV curve of titanium mesh in the E3 electrolyte at a scan rate of 10 mV s^-1^

**Figure S3** displays CV curves of titanium meshes in the E3 electrolytes. During cathodic scanning, the current density sharply increases at -0.72V, which corresponds to the zinc platting. During anodic scanning, a redox peak at about -0.4 V vs. Ag/AgCl can be ascribed to the zinc stripping, while the current density significantly increases at 1.4 V vs. Ag/AgCl can be assigned to the oxidation of chloride ions.


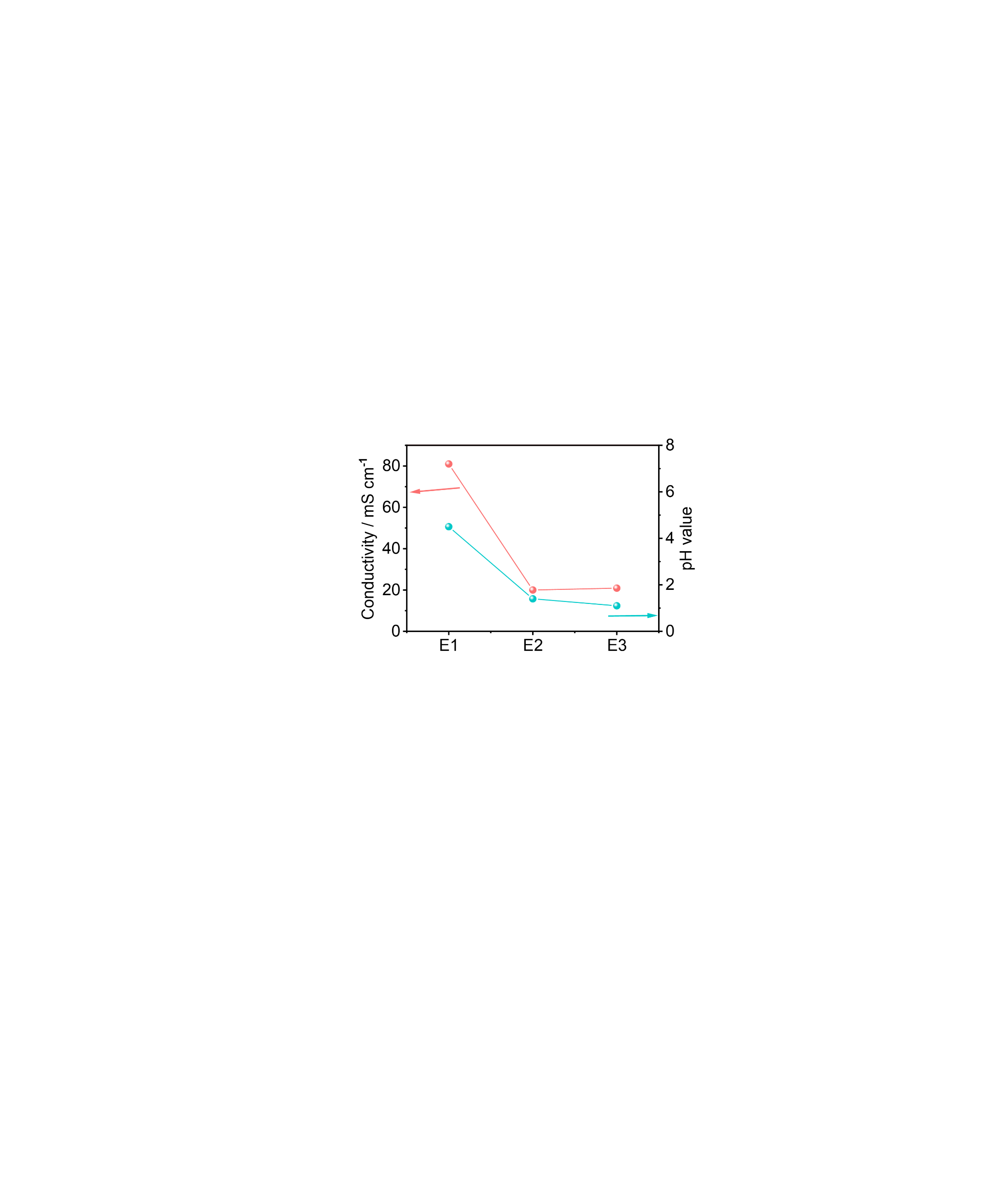


**Fig. S4** pH values and ionic conductivities of different electrolytes


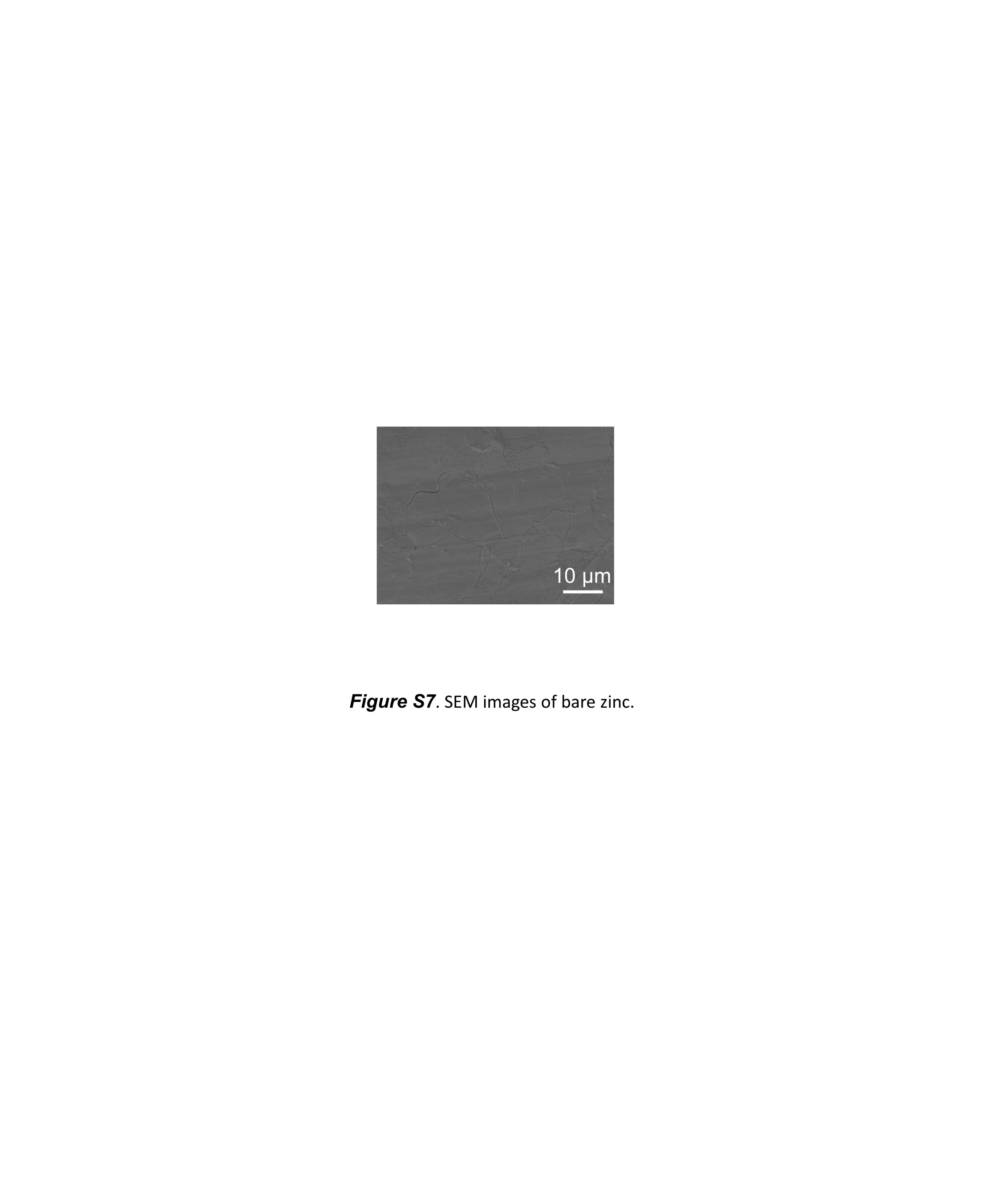


**Fig. S5** Top-view SEM image of bare zinc foil


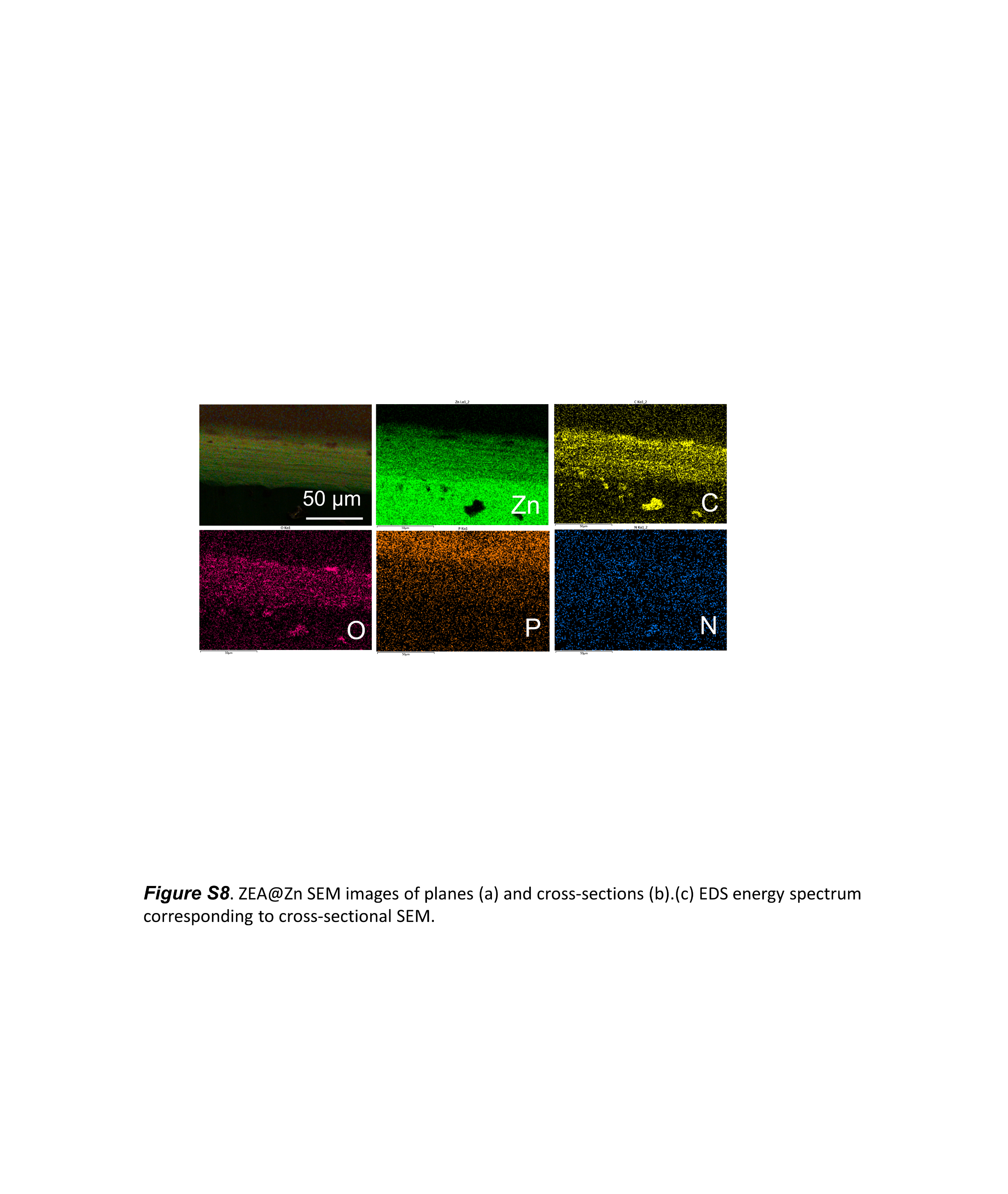


**Fig. S6** Elemental mapping of Zn, C, O, P, N in the ZEA@Zn metal anode

In **Fig. S6**, we also notice that the N elemental mapping lack a clear regional distribution. The first possible reason may be the relatively low content of nitrogen element in the sample. Additionally, a small amount of N_2_ in the air can absorb on the sample, which causes the contamination of samples, therefore leading to an unclear elemental distribution.


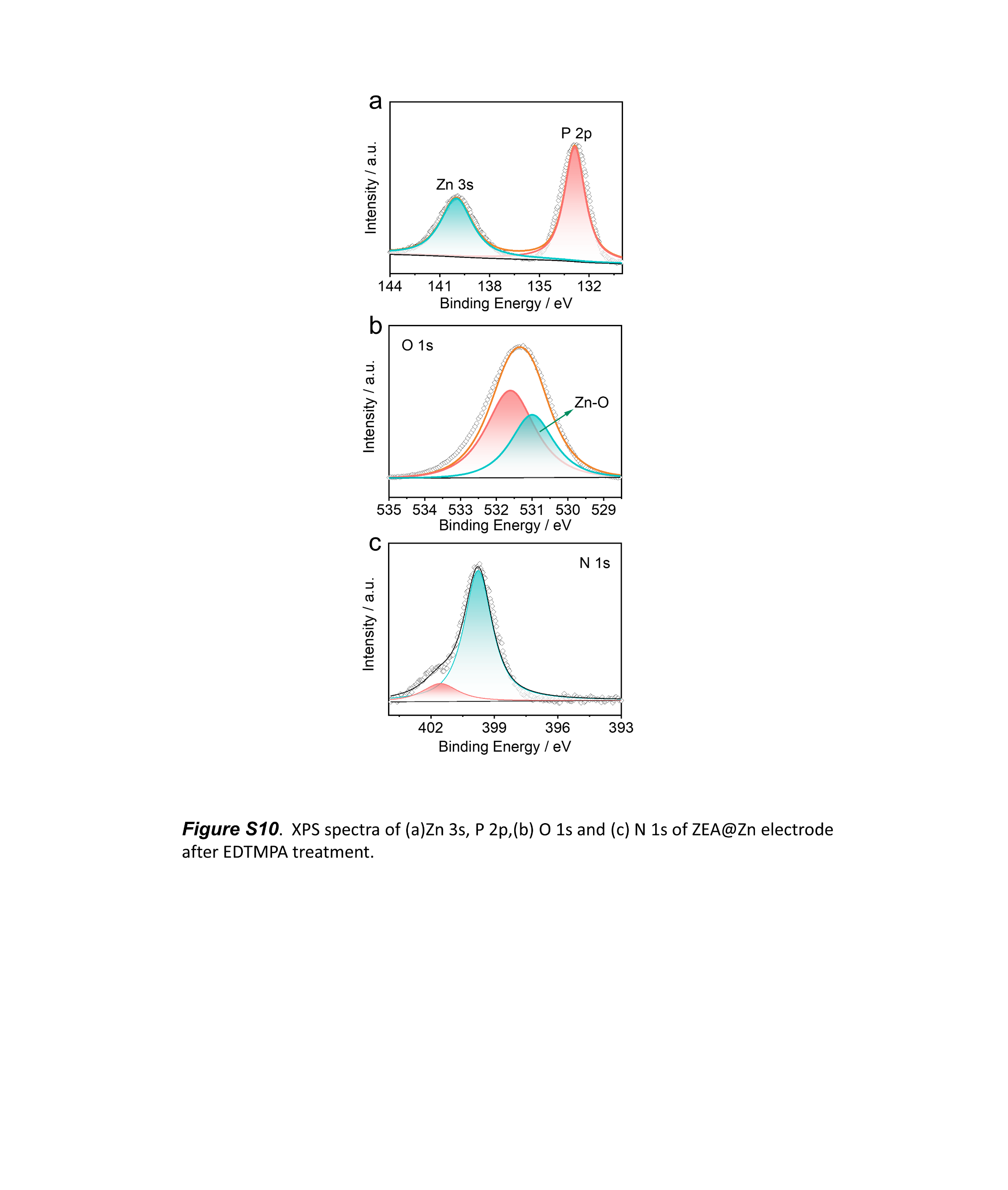


**Fig. S7** XPS spectra of (**a**) Zn 3s and P 2p, (**b**) O 1s, and (**c**) N 1s of the ZEA@Zn metal anode

As shown in **Fig. S7**, the peak at ~140 eV is attributed to the Zn 3s, the P 2p peak located at 132.9 eV corresponds to PO_4_^3-^, which is also consistent with FTIR results [S10]. In O 1s XPS, a peak at 531 eV was detected, which can be ascribed to the Zn-O bonds of the ZEA coordination compound [S11]. In the N 1s XPS, the peaks at 401.6 eV and 399.8 eV demonstrate that N atoms exist in two chemical states: N-C and N-H^+^ (N atoms coordinate with H^+^ in solution) [S11, S12].


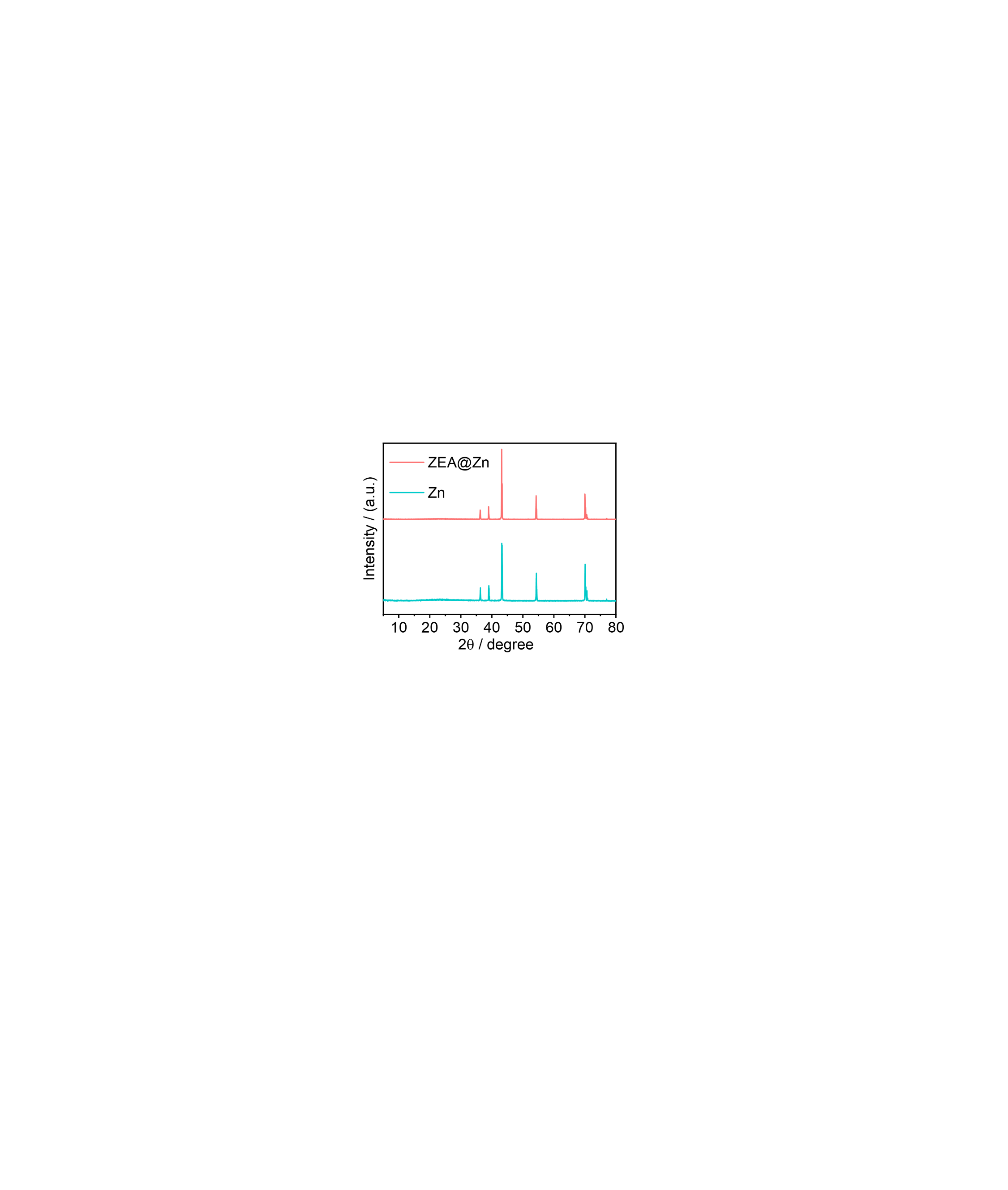


**Fig. S8** XRD patterns of bare Zn and ZEA@Zn metal anodes


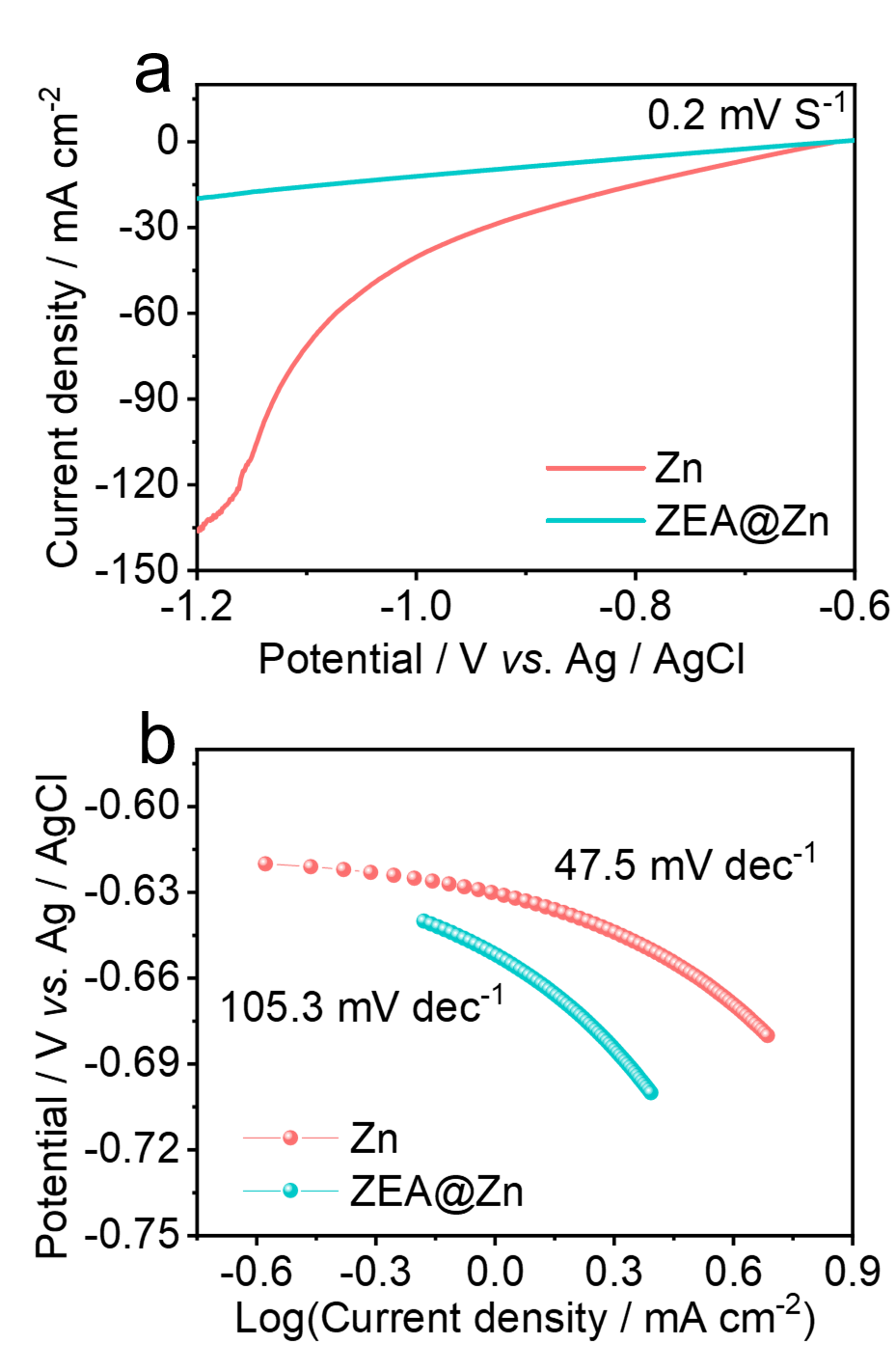


**Fig. S9** (**a**) Linear sweep voltammetry curves of bare Zn and ZEA@Zn anodes in the E3 electrolyte at 0.2 mV s^-1^. (**b**) The corresponding Tafel slopes of bare Zn and ZEA@Zn anodes


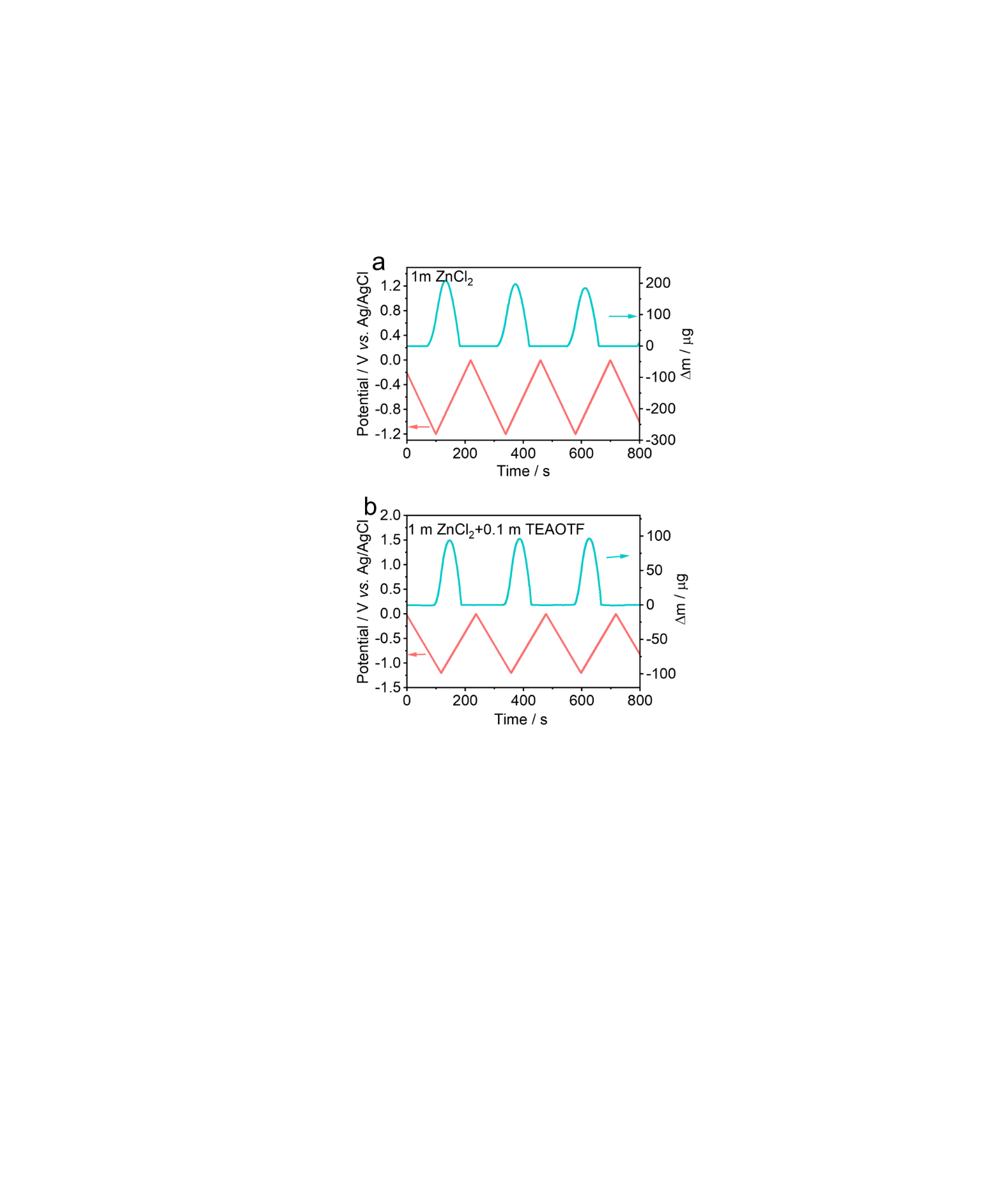


**Fig. S10** The plot for Sauerbrey mass and potential as functions of time during CV measurements at 10 mV s^-1^ in (a) 1 m ZnCl_2_ and (b) 1 m ZnCl_2_+0.1 m TEAOTf electrolytes


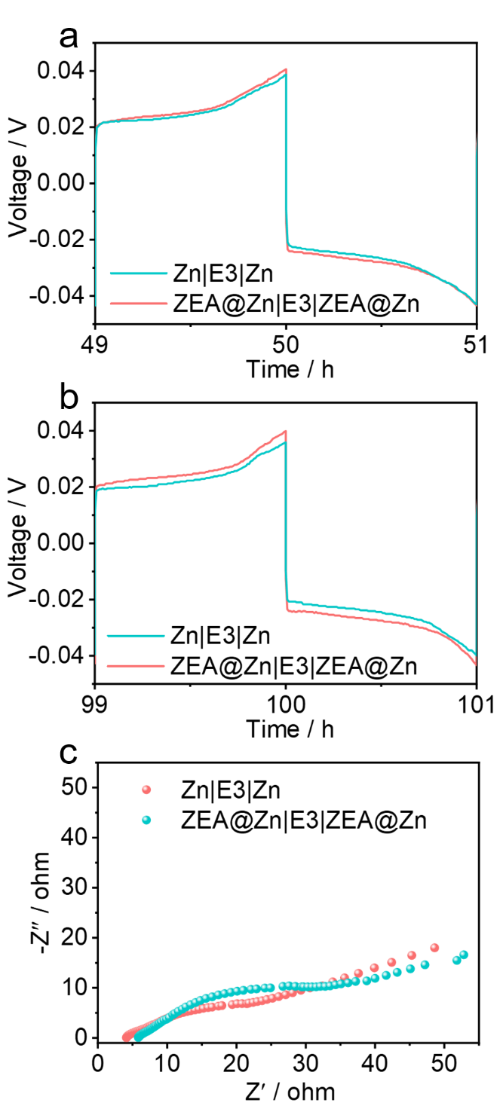


**Fig. S11** The voltage profiles of different symmetric cells at (**a**) 50 hours and (**b**) 100 hours. (**c**) EIS curves of Zn|E3|Zn and ZEA@Zn|E3|ZEA@Zn symmetric cells after 2 cycles

As shown in the voltage profiles of symmetric cells (**Fig. S11a-b)**, it can be observed that the ZEA@Zn|E3|I_2_@CC cell exhibits relatively larger polarization compared with the Zn|E3|I_2_@CC cell. Additionally, the EIS curves (shown in **Fig. S11c**) confirms that the ZEA-coated Zn anode possess slightly enhanced interphase resistance.


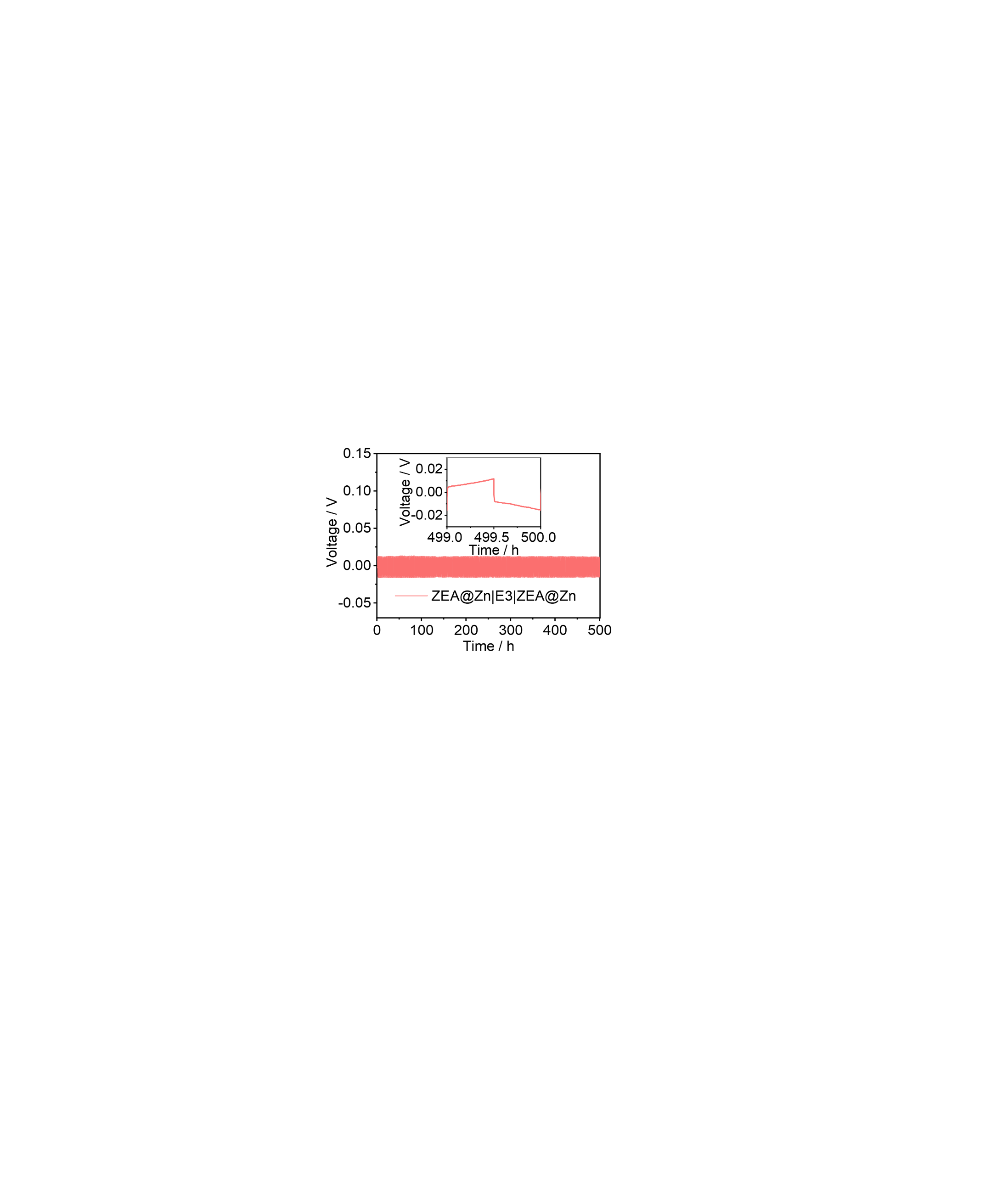


**Fig. S12** The cycling stability of ZEA@Zn|E3|ZEA@Zn symmetric cells (0.5 h charge-0.5 h discharge with the current density of 0.5 mA cm^-2^)


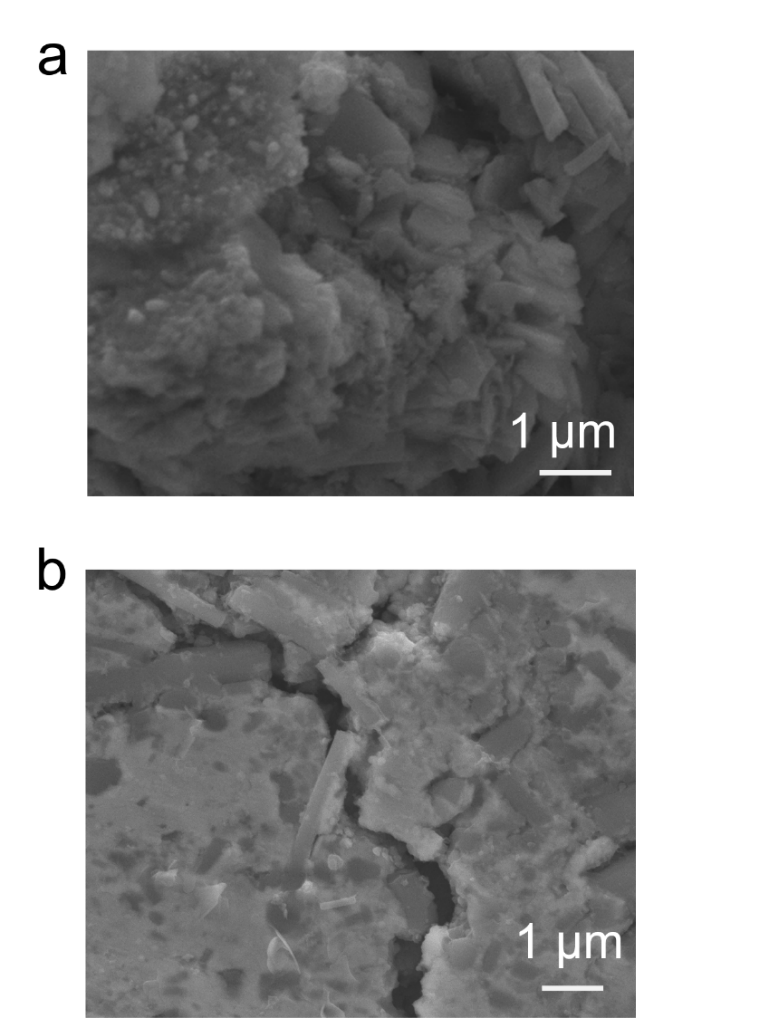


**Fig. S13** The top-view SEM images of Zn metal anodes after cycling in the (**a**) E2 and (**b**) E3 electrolytes


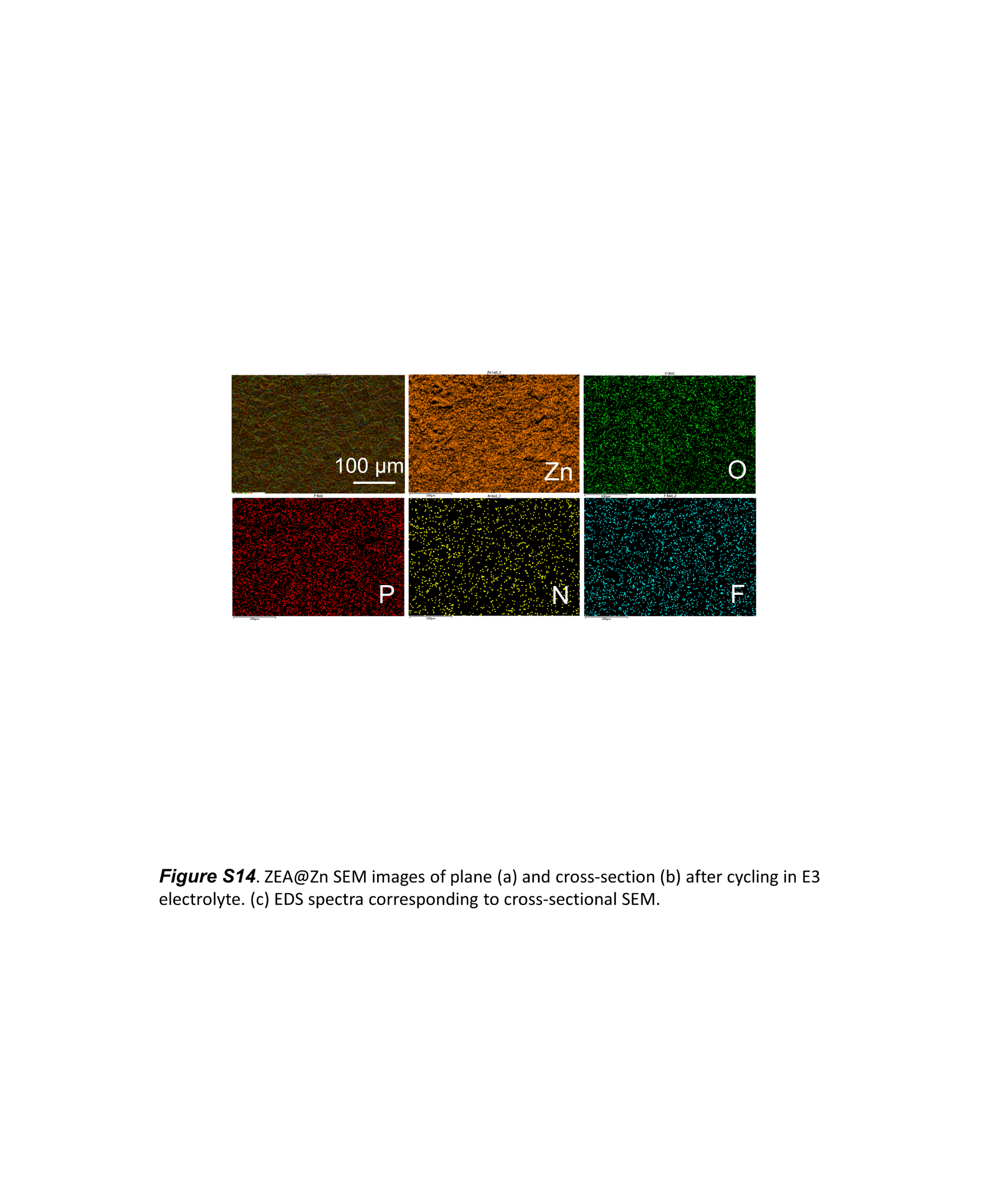


**Fig. S14** Elemental mapping of Zn, O, P, N, F of the cycled ZEA@Zn in the E3 electrolyte


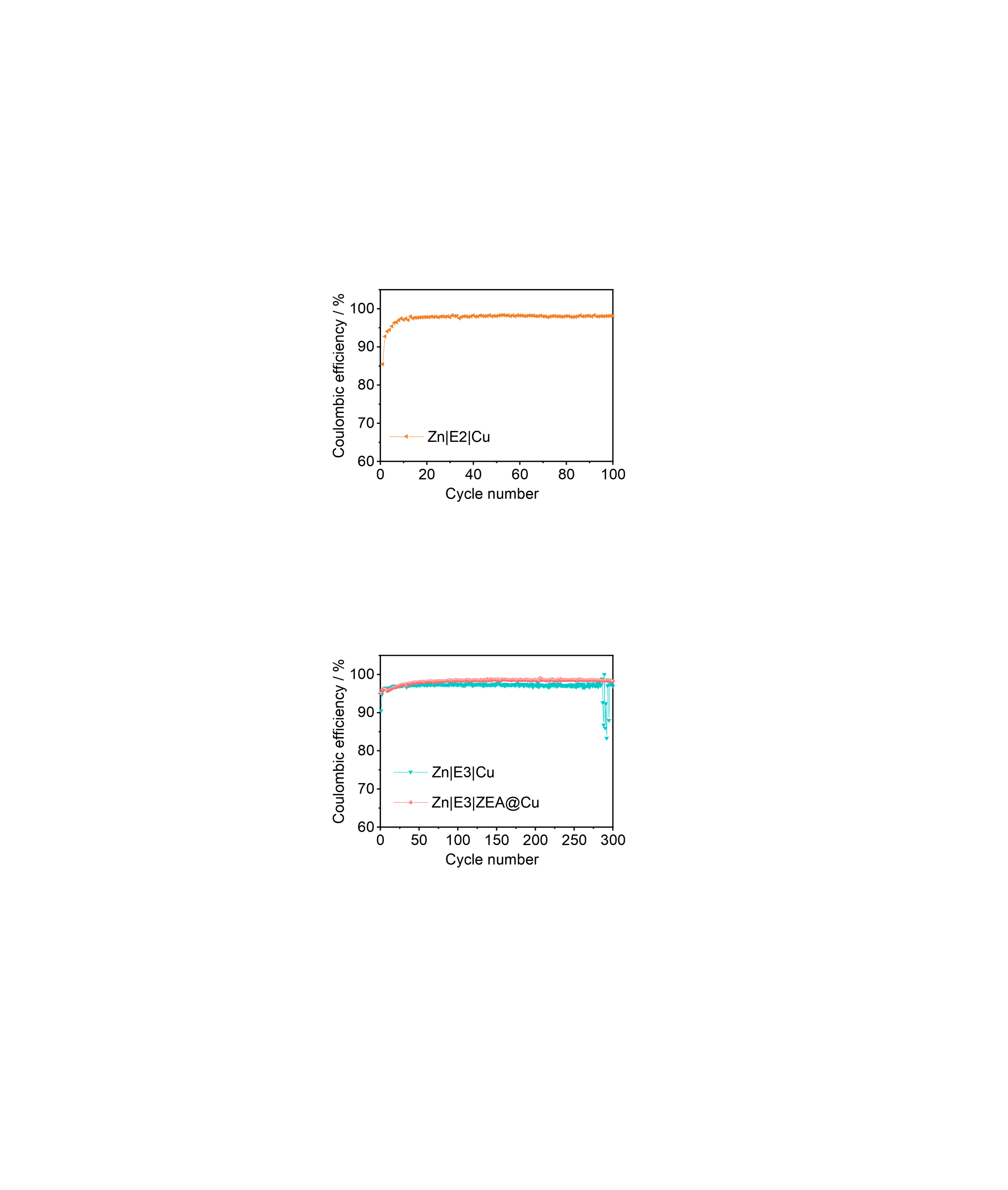


**Fig. S15** Coulombic efficiencies of Zn plating/stripping on bare Cu in the E2 electrolyte with a current density of 0.5 mA cm^-2^ and plating capacity of 0.5 mAh cm^-2^


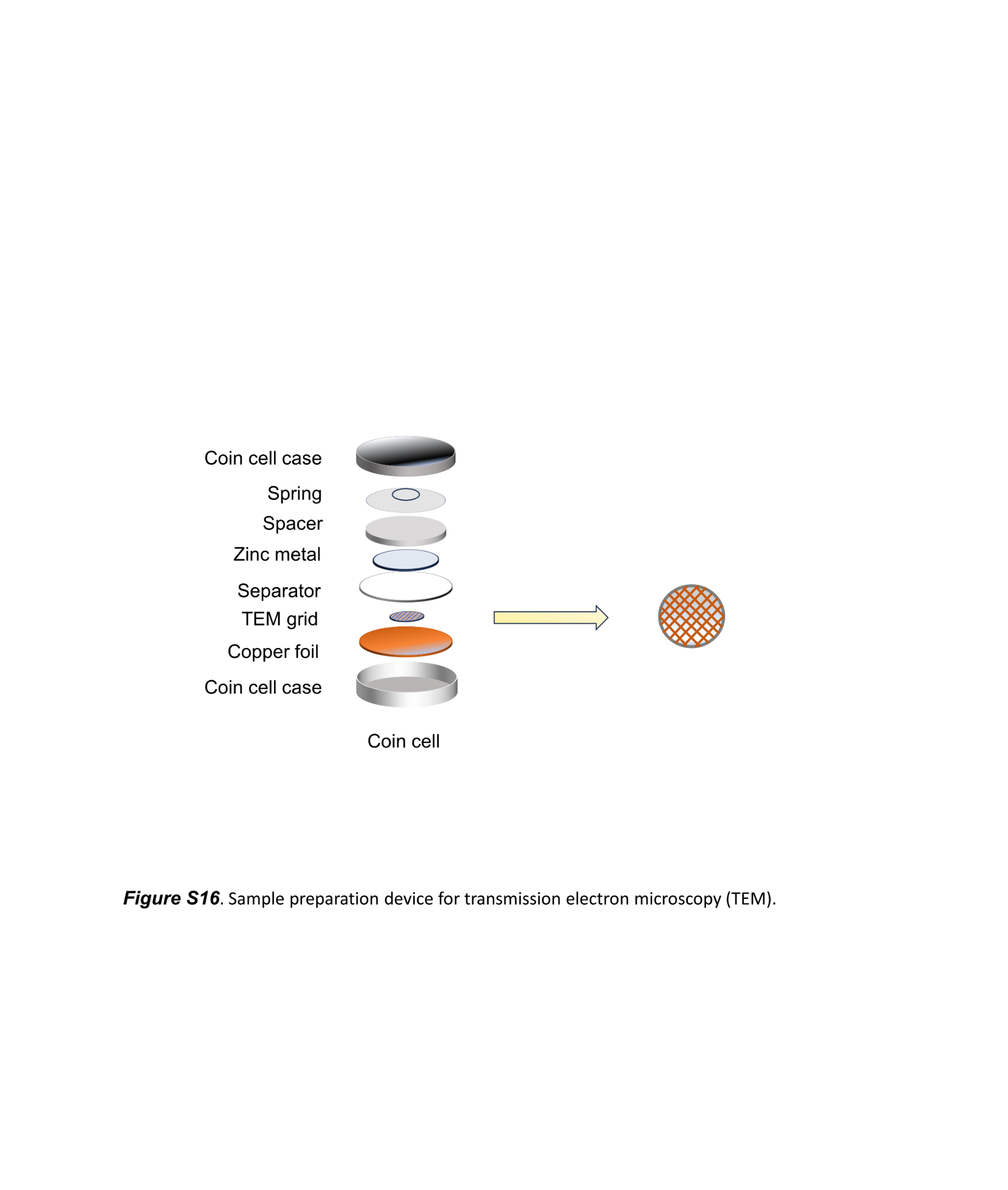


**Fig. S16** Schematic illustration showing the preparation of the double-layered architecture on TEM grid


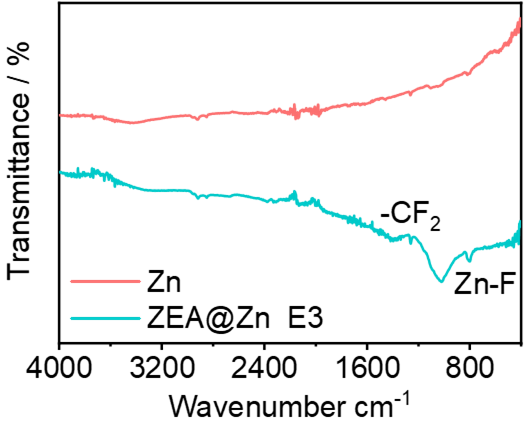


**Fig. S17** FTIR spectra of bare zinc metal anode and cycled ZEA@Zn anode in the E3 electrolyte

By cycling the ZEA@Zn in the E3 electrolyte, the vibration peaks of 802 cm^-1^ and 1263.8 cm^-1^ can be observed in the FTIR spectrum, which can be ascribed to the Zn-F and -CF_3_, respectively (**Fig. S17)** [S13, S14].


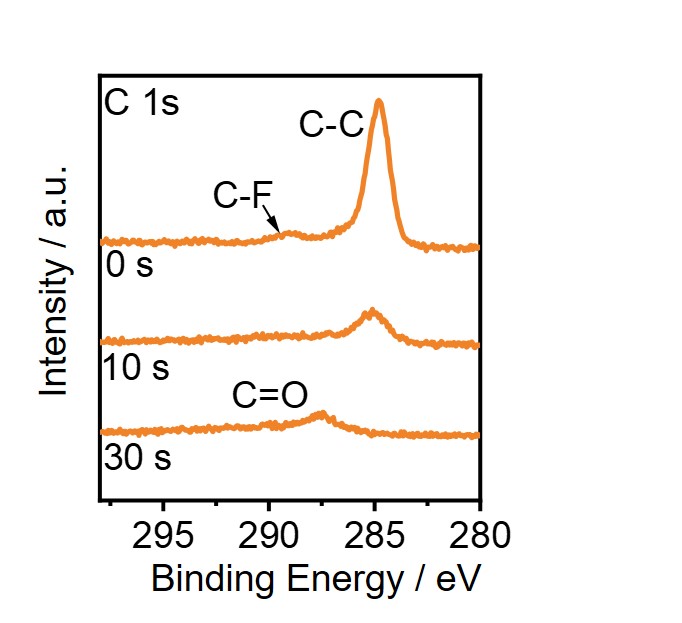


**Fig. S18** The in-depth C 1s XPS spectra of the cycled ZEA@Zn anode


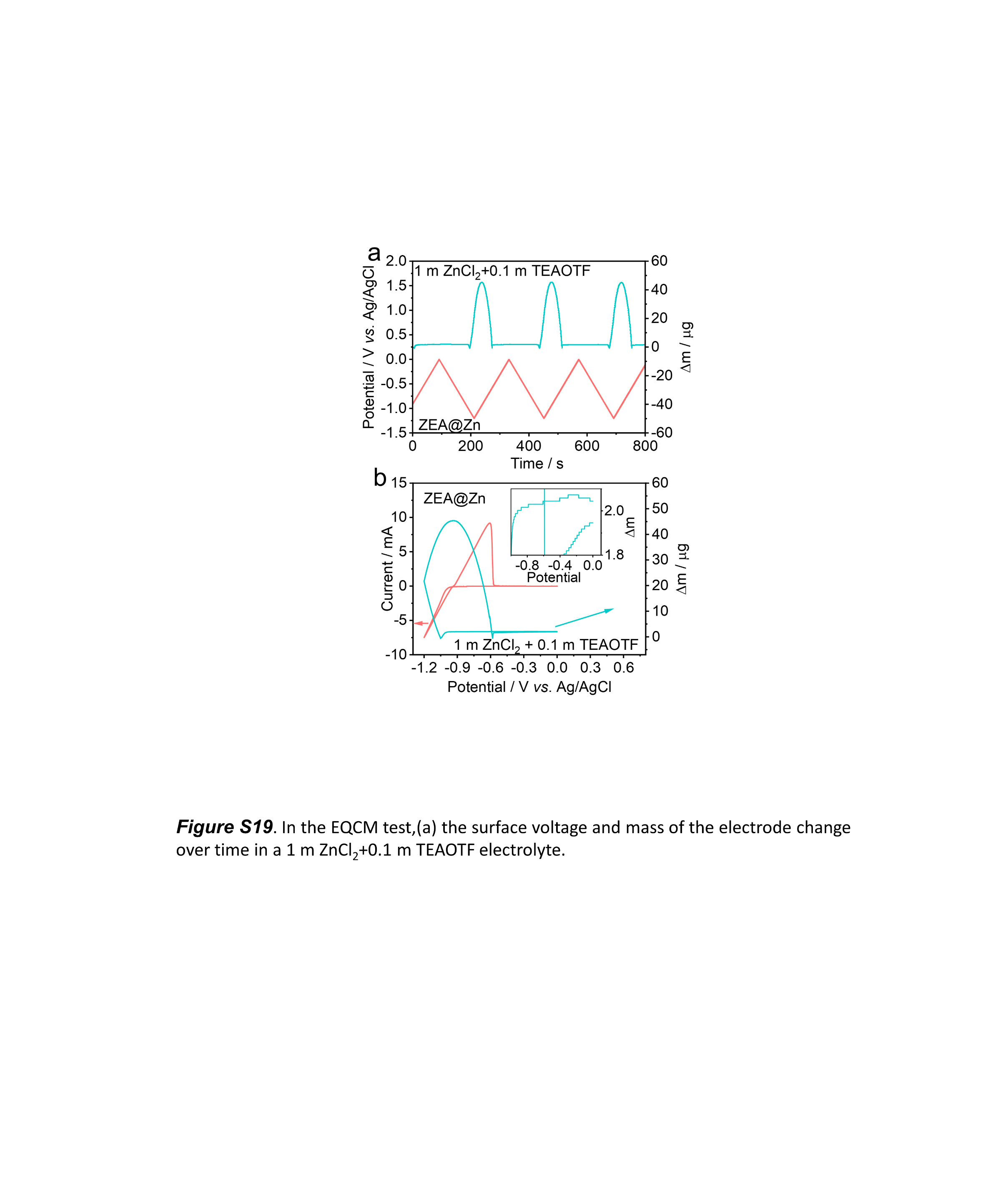


**Fig. S19** (**a**) The plot for Sauerbrey mass and potential of ZEA@Zn as functions of time in 1 m ZnCl_2_+0.1 m TEAOTf electrolyte. (**b**) The CV curve and the corresponding mass evolution of ZEA@Zn in 1 m ZnCl_2_+0.1 m TEAOTf electrolyte


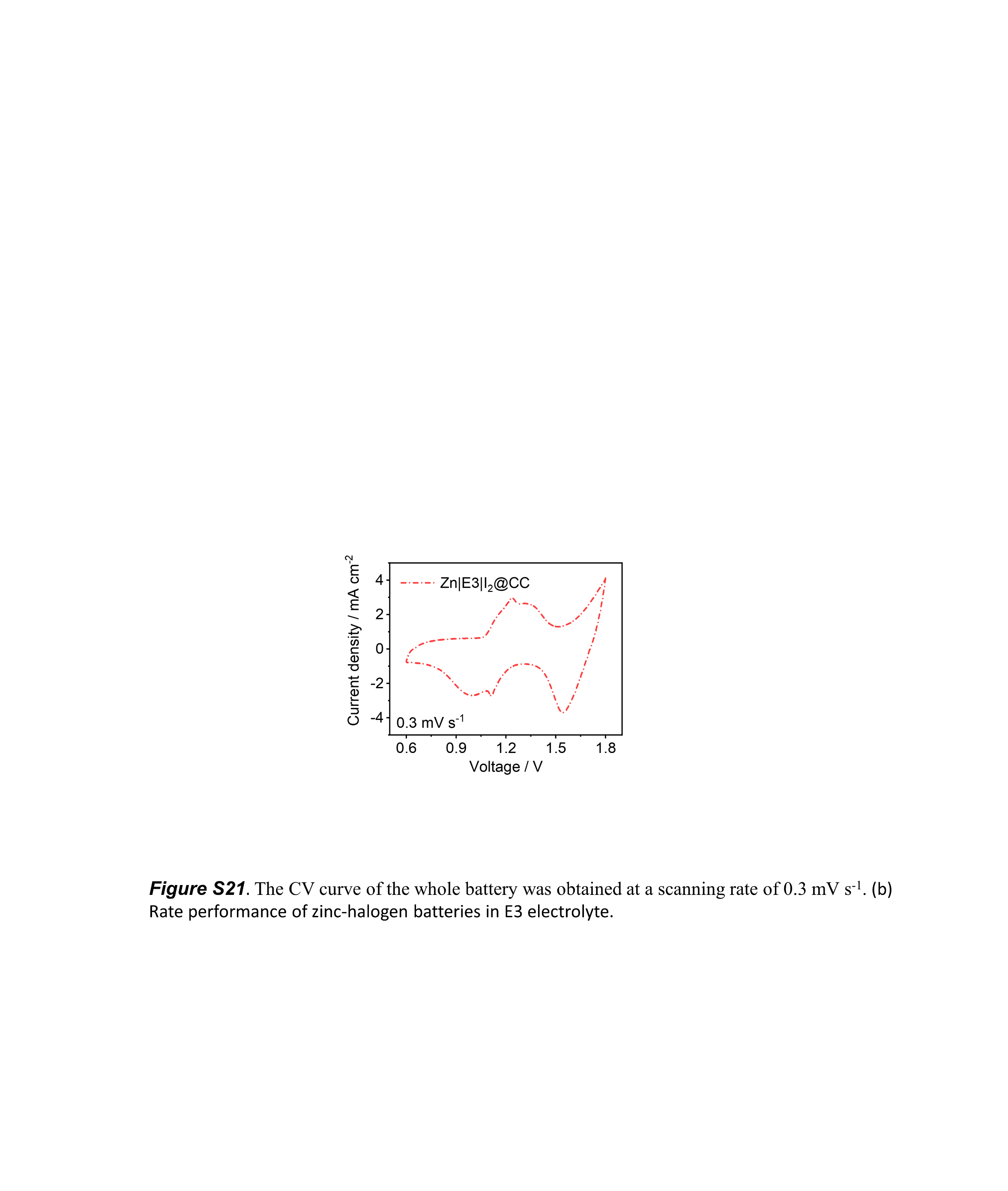


**Fig. S20** CV curve of the aqueous zinc-iodine battery with the E3 electrolyte at a scan rate of 0.3 mV s^-1^


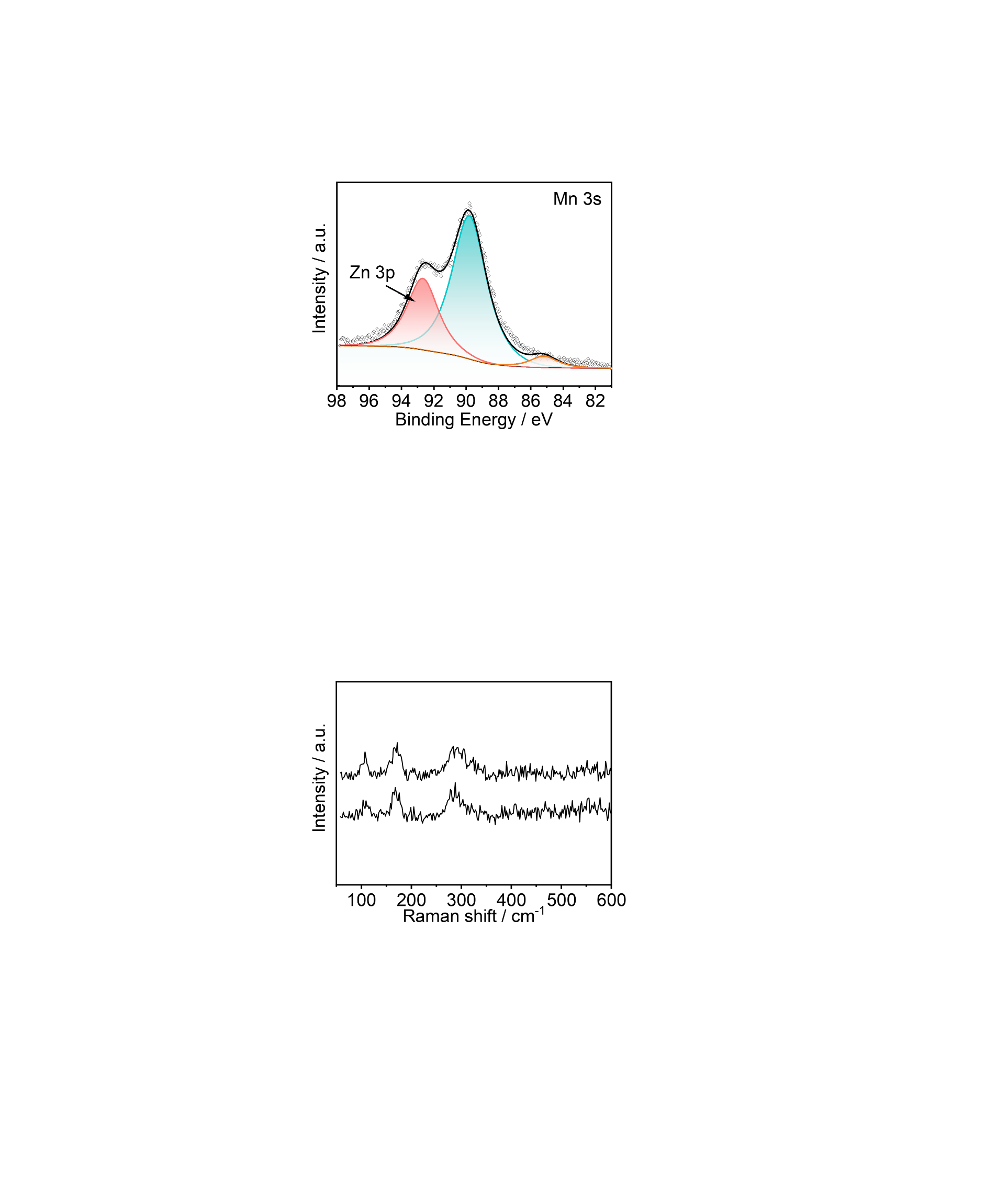


**Fig. S21** The high-resolution XPS spectrum of Mn 3s of the charged cathode

According to the literature [S15], the redox potential of Mn^4+^/Mn^2+^ under acidic environment can be calculated by the Nernst equation (Eq. S7):

E = 1.23 − 0.0296 lg[Mn^2+^] + 0.1184lg[H^+^] (S7)

Obviously, the Mn^4+^/Mn^2+^ redox reactions can occur with the generation of MnO_2_ as the product during the charging process of the aqueous zinc-based batteries (voltage window from 0.6~1.8 V).

To identify the peaks at 1.22/1.11 V, we have further conducted XPS measurements on charged cathode. As shown in **Fig. 21**, a Mn 3s peak can be observed to locate at 89.8 eV and 85 eV, while a Zn 3p peak is situated at 92.8 eV, which indicates the insertion of zinc ions into the interlayer of MnO_2_ according to the Eq. S8. This result is highly consistent with the previously reported literatures [S16].

MnO_2_ + 0.5 Zn^2+^ + e^-^ $\leftrightarrow$ Zn_0.5_MnO_2_ (S8)


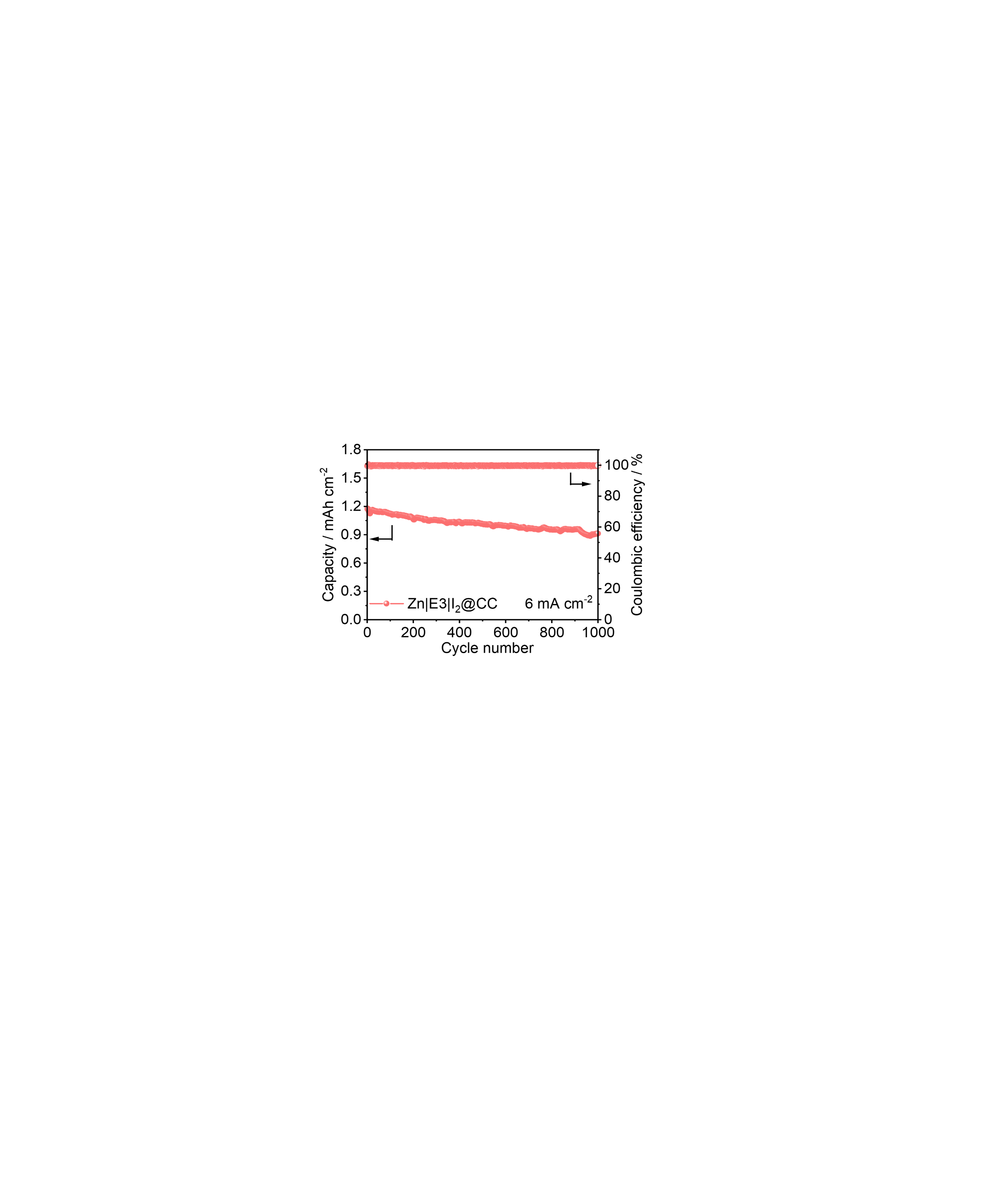


**Fig. S22** Cycling performance of Zn|E3|I_2_@CC cell at a current density of 6 mA cm^-2^


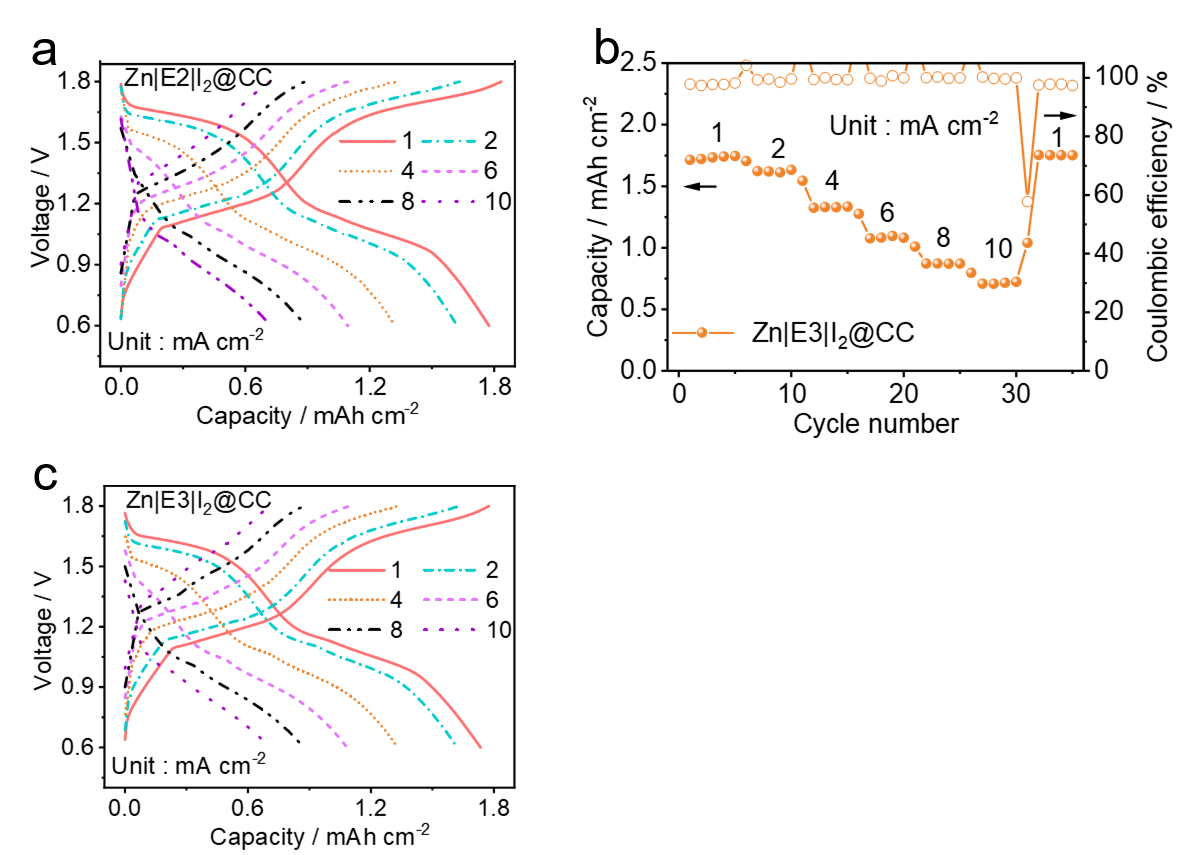


**Fig. S23** (**a**) The voltage profiles of the Zn|E2|I_2_@CC cell at different current densities. (**b**) Rate performance of the Zn|E3|I_2_@CC cell at different current densities. (**c**) The corresponding voltage profiles of the Zn|E3|I_2_@CC cell at different current densities


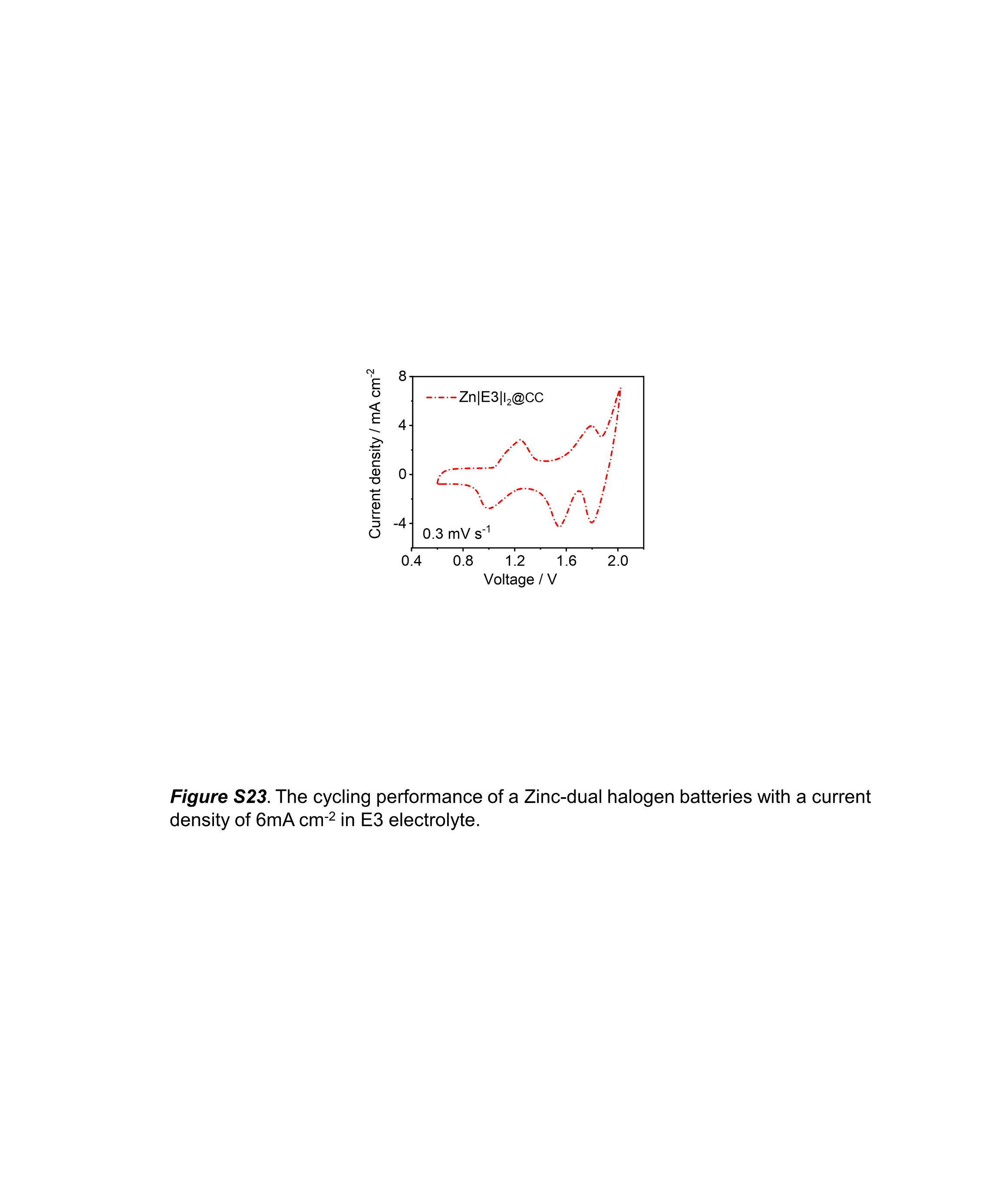


**Fig. S24** CV curve of the aqueous zinc-dual halogen battery with the E3 electrolyte at a scan rate of 0.3 mV s^-1^


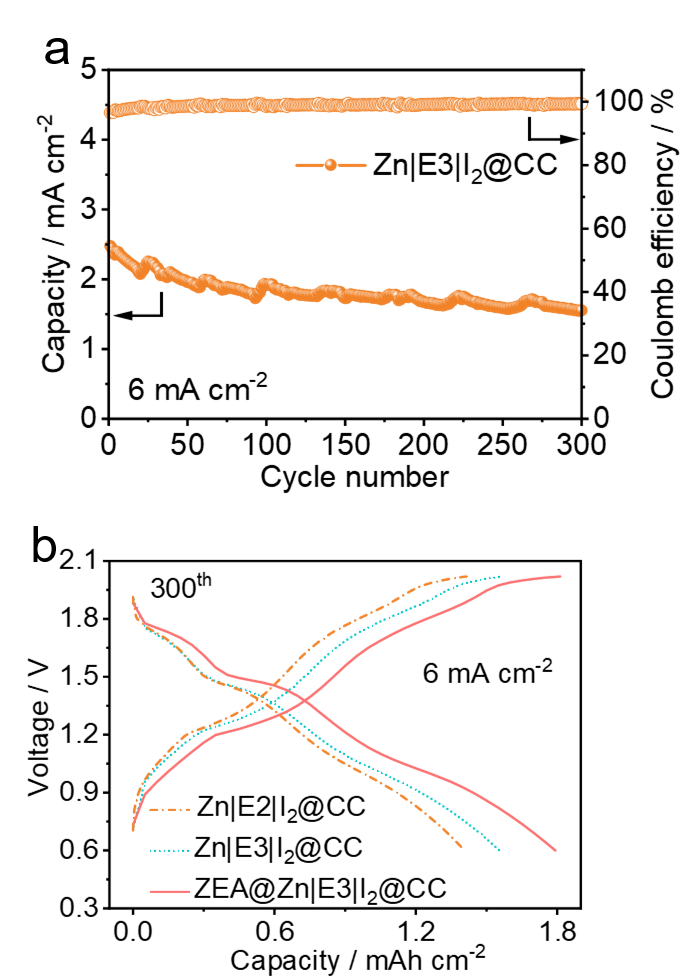


**Fig. S25** (**a**) Cycling performance of aqueous zinc-dual halogen battery (Zn|E3|I_2_@CC cell) at a current density of 6 mA cm^-2^. (**b**) The corresponding voltage profiles of aqueous zinc-dual halogen batteries at 300 cycles


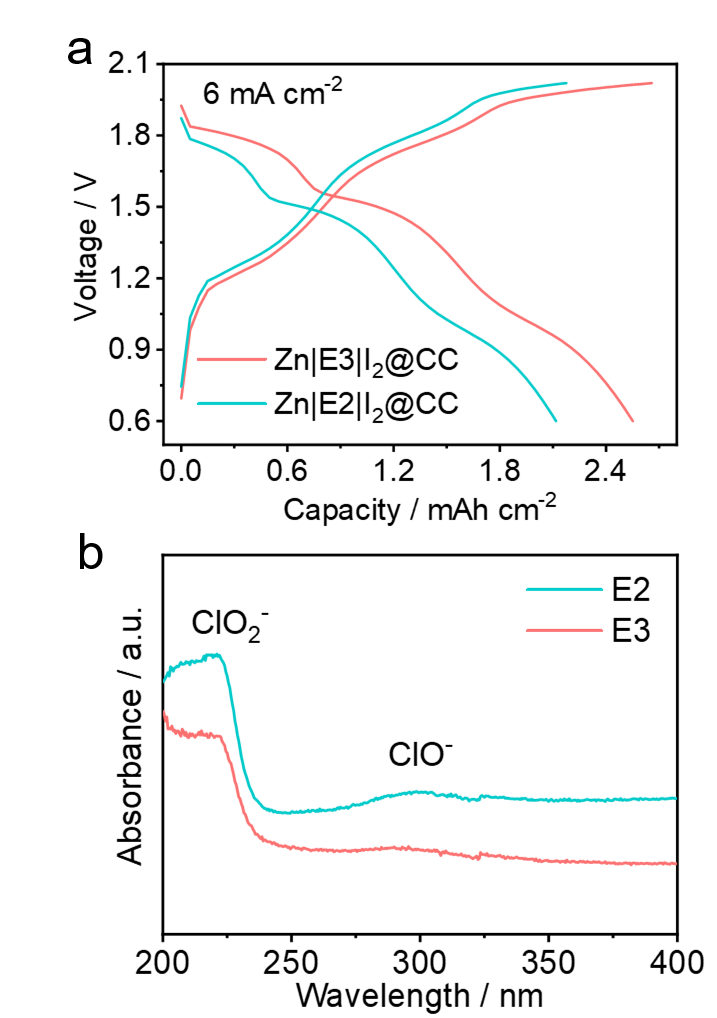

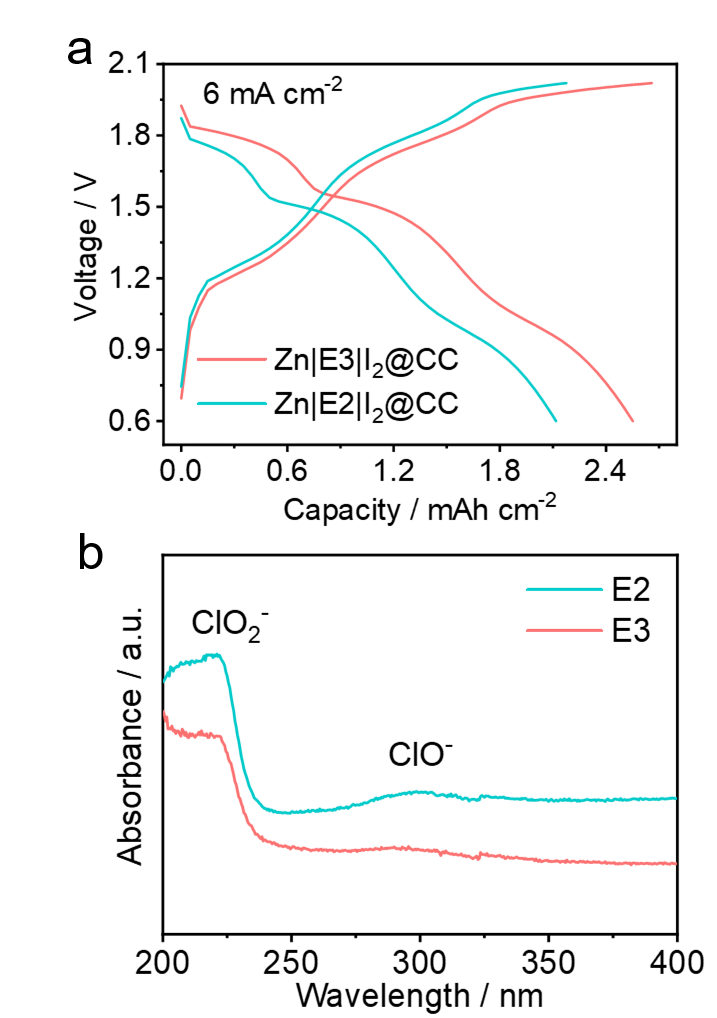


**Fig. S26** (**a**) The corresponding voltage profiles of Zn|E2|I_2_@CC and Zn|E3|I_2_@CC aqueous zinc-dual halogen batteries. (**b**) UV-vis spectra of cycled electrolytes

Ultraviolet-visible (UV-vis) spectroscopy was performed to confirm the adsorption effect of MnO_2_ towards active species. We assembled three-electrode configuration with carbon cloth working electrode and E2/E3 electrolytes. Then, UV-vis spectroscopy has been applied to investigate the cycled electrolytes (**Fig. S26b**). In the E2 electrolyte, chlorinated by-products (ClO^-^ and ClO_2_^-^) can be observed at 300.2 and 221.5 nm in the UV-vis spectrum, while these signals are almost diminishing in the E3 electrolyte. This again confirms the MnO_2_ can restrain the active species [S17, S18].


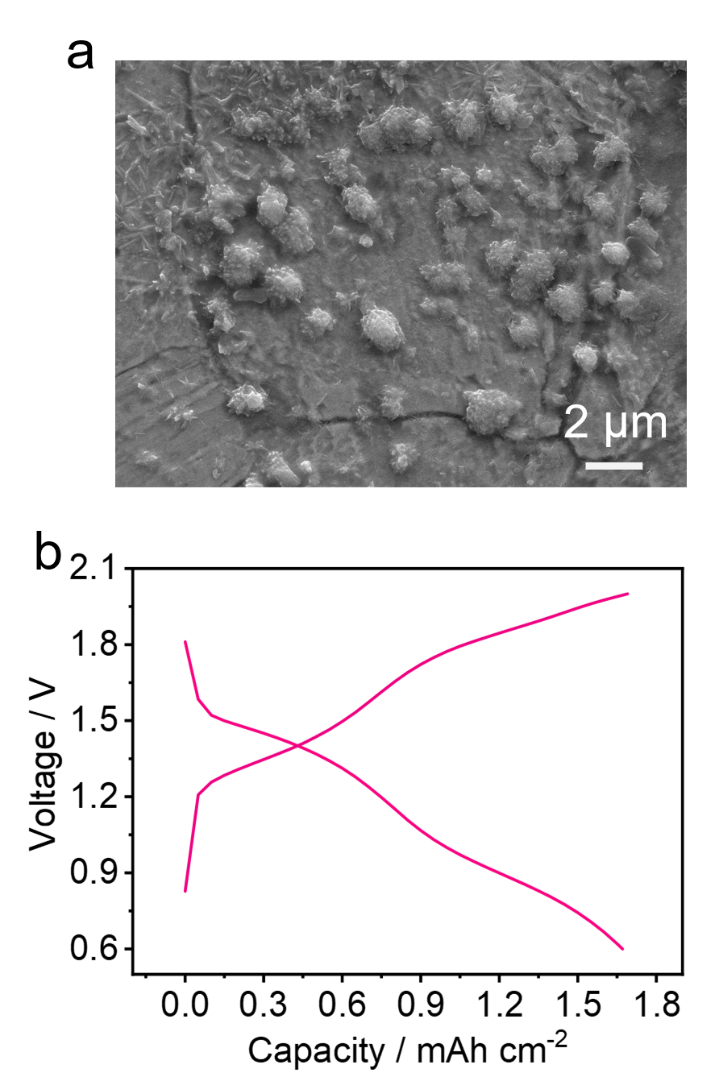


**Fig. S27** (**a**) SEM image of the zinc metal anode after etching 40 minutes. (**b**) The corresponding voltage profile with zinc metal anode after etching 40 minutes

As shown in **Fig. S27a**, extension of etching time results in an uneven surface with cracks on the metal anode. Meanwhile, **Fig. S27b** exhibits the voltage profile with zinc metal anode after etching 40 minutes, in which the huge battery polarization leads to deceased capacity (1.67 mAh cm^-2^).


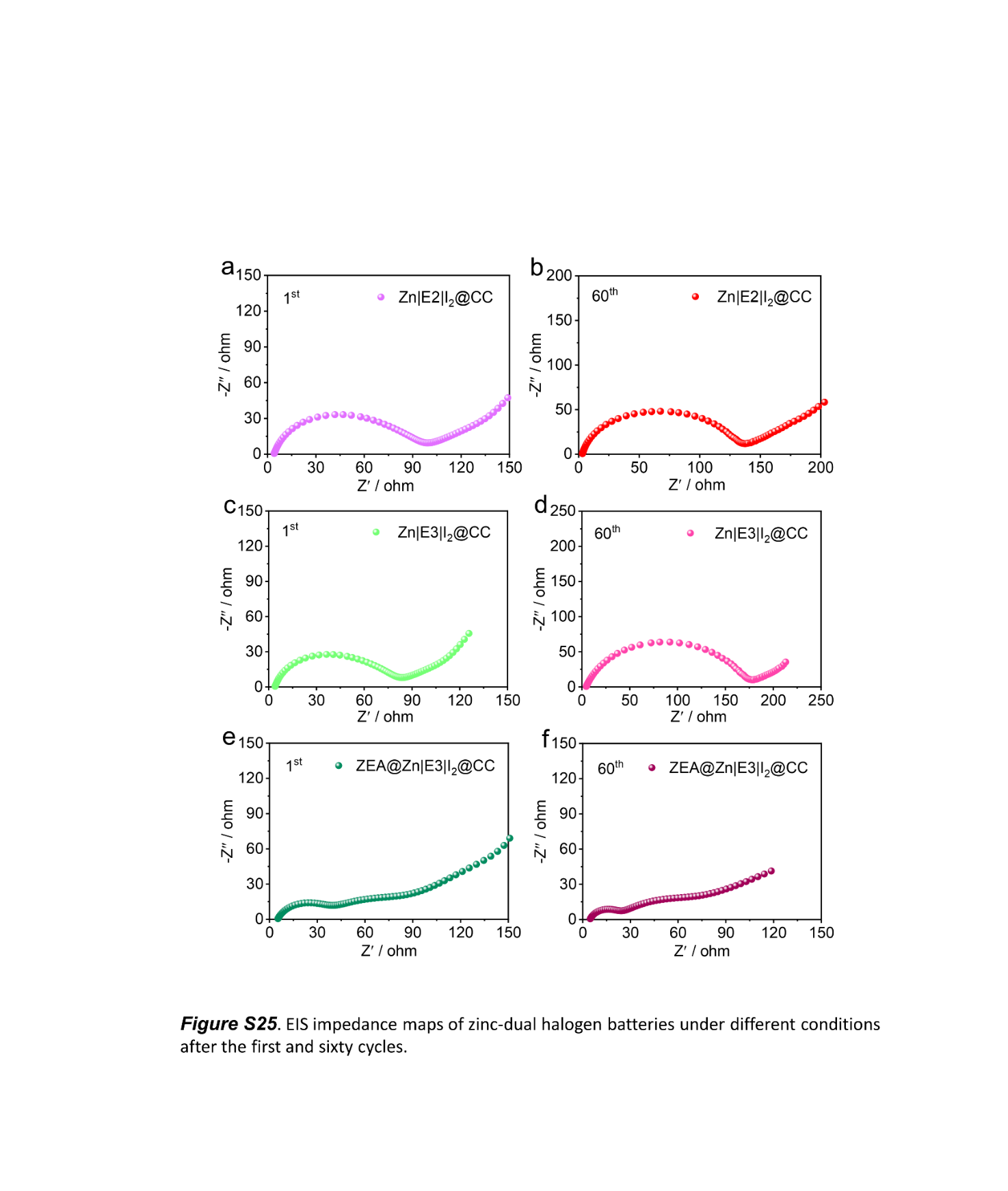


**Fig. S28** EIS curves of aqueous zinc-dual halogen batteries after the first and 60 cycles

**
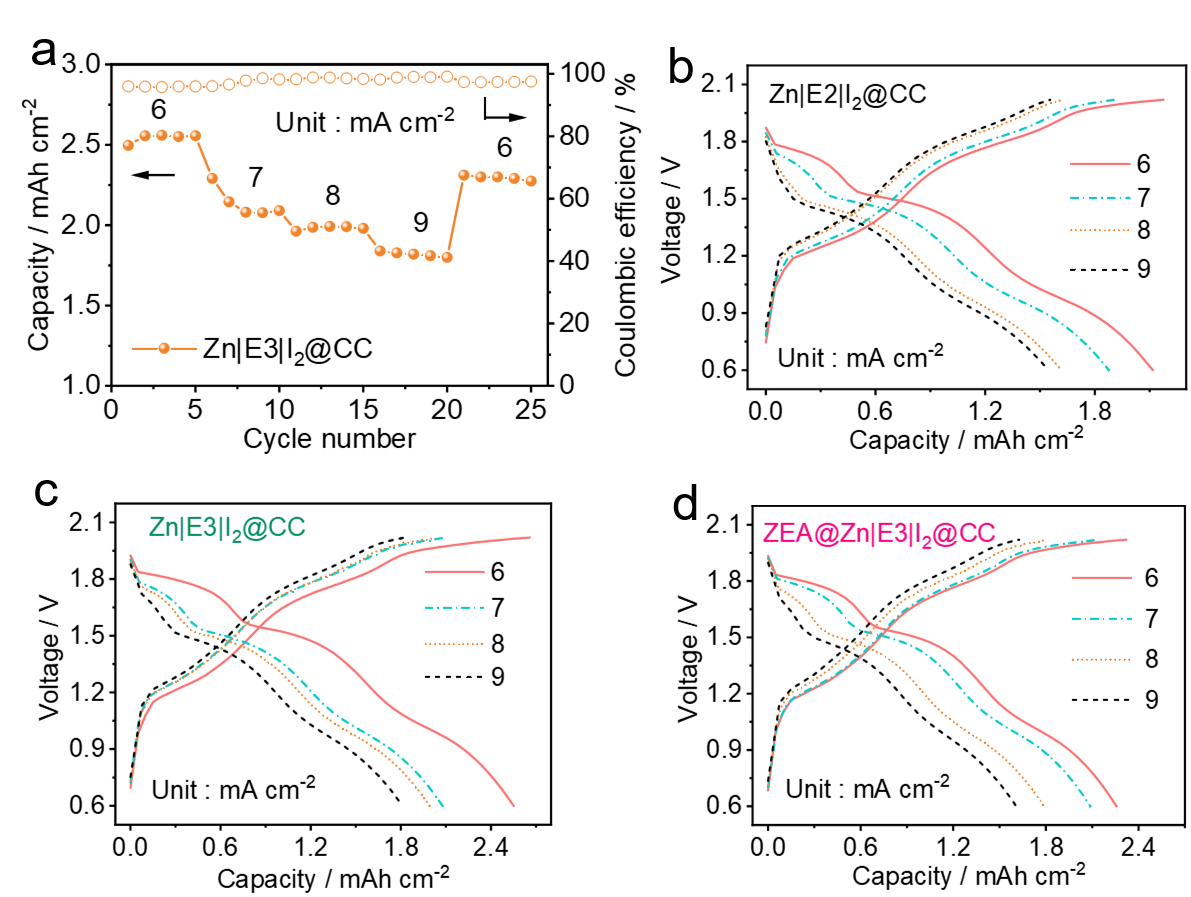
**

**Fig. S29** (**a**) Rate performance of the aqueous zinc-dual halogen battery (Zn|E3|I_2_@CC cell). The corresponding voltage profiles of (**b**) Zn|E2|I_2_@CC, (**c**) Zn|E3|I_2_@CC, and (**d**) ZEA@Zn|E3|I_2_@CC aqueous zinc-dual halogen batteries at different current densities


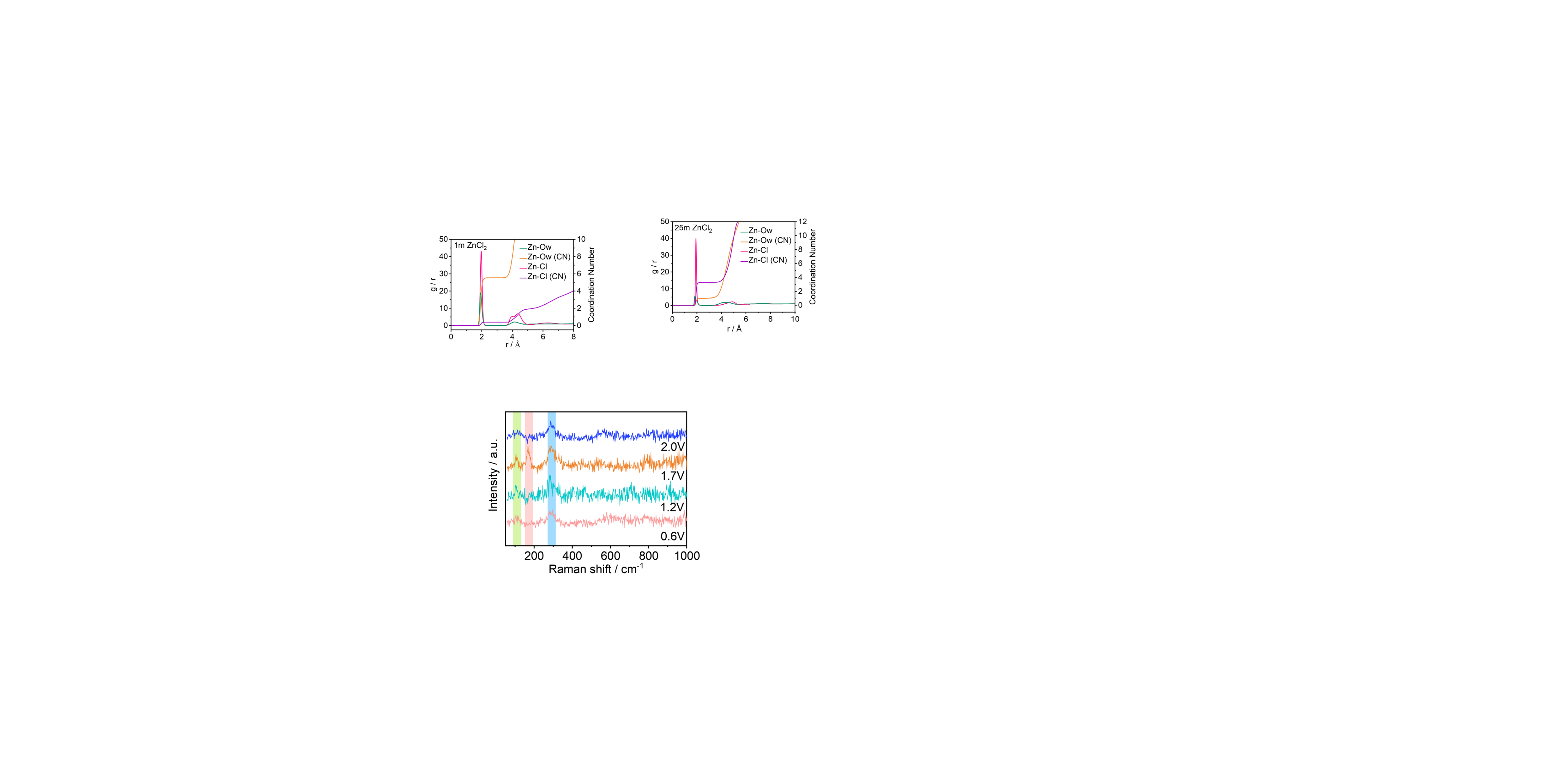


**Fig. S30** *ex situ* Raman spectra of I_2_@CC cathode at different charge states

To further characterize the reaction, we have performed the ex situ Raman measurements on I_2_@CC cathode at different charging states. As shown in **Fig. S30**, the peak at ~290 cm^-1^ can be ascribed to the Cl^-^ coordinating Zn^2+^ species adsorbed on the electrode as hydrated [ZnCl^2+x^(H_2_O)_y_]^x-^ in the electrolyte [S19], while the peaks at 105 and 172 cm^-1^ correspond to I_3_^-^ [S20] and [ICl_x_]^1-x^ (x = 1, 2, etc.) [S21], respectively. During the charging process, the I_3_^-^ signal emerges when charging to 1.2 V, while the [ICl_x_]^1-x^ peak appears after charging to 1.7 V, which confirms the I^0^/I^-^ and I^+^/I^0^ redox reactions at lower-ordered plateaus. However, when charging the aqueous zinc-dual halogen batteries to 2.0 V, both I_3_^-^ and [ICl_x_]^1-x^ signals disappear, which suggests the generations of new active species.

**Table S1** Comparison of electrochemical performance with various batteries [S22-S32]

| Battery systems | Conductive ion | Voltage (V) | Capacity (mAh cm^-2^) | Refs. |
| --- | --- | --- | --- | --- |
| Zn\|\| PAC-I_2_ | Zn^2+^ | $1.25$ | 2 | [S22] |
| Zn\|\|Ti_3_C_2_I_2_MXene | Zn^2+^ | $1.35$ | 0.26 | [S23] |
| COF-Zn \|\| Br_2_-exCOF | Zn^2+^ | $1.58$ | 0.86 | [S24] |
| Zn \|\| Zn_0.25_V_2_O_2_ | Zn^2+^ | $0.81$ | 1.69 | [S25] |
| Zn \|\| MnO_2_ | Zn^2+^ | $1.35$ | 0.86 | [S26] |
| Zn \|\| CNT/VOOH | Zn^2+^ | $0.9$ | 0.72 | [S27] |
| Li_4_Ti_5_O_12_ \|\| LiMn_2_O_4_ | Li^+^ | $2.3$ | 0.24 | [S28] |
| Zn/GFF \|\| PGA | Zn^2+^ | $1.5$ | 1.69 | [S29] |
| Zn \|\| Od-HVO@PPy | Zn^2+^ | $0.8$ | 0.6 | [S30] |
| Zn \|\| S | Zn^2+^ | $0.4$ | 0.77 | [S31] |
| S/C \|\| I_2_@CC  Zn \|\| I-Cl/CC | Li^+^  Zn^2+^ | $1.24$  1.42 | 0.96  2.3 | [S32]  This work |

**Table S2** Compositions of simulated electrolytes

|  | E1 | E2 | E3 |
| --- | --- | --- | --- |
| Number of H_2_O per box | 11000 | 11000 | 11000 |
| Number of ZnCl_2_ per box | 200 | 5000 | 5000 |
| Number of MnSO_4_ per box  Number of TEAOTf per box | **/**  **/** | **/**  **/** | 1  20 |
| T(K) | 298 | 298 | 298 |

**Supplementary References**

1. S. Plimpton, Computational limits of classical molecular dynamics simulations. Comput. Mater. Sci. **4**, 361–364 (1995). <https://doi.org/10.1016/0927-0256(95)00037-1>
2. L. Martínez, R. Andrade, E.G. Birgin, J.M. Martínez, PACKMOL: a package for building initial configurations for molecular dynamics simulations. J. Comput. Chem. **30**, 2157–2164 (2009). <https://doi.org/10.1002/jcc.21224>
3. Y.-Y. Li, M. Wang, C.-C. Wang, Q.-W. Zhang, H.-B. Yi, Distinctive hydration dynamics around highly coordinated Cu^2+^/Zn^2+^-chloride complexes: a molecular dynamics simulation study. J. Mol. Liq. **314**, 113619 (2020). <https://doi.org/10.1016/j.molliq.2020.113619>
4. C.F. Schwenk, H.H. Loeffler, B.M. Rode, Structure and dynamics of metal ions in solution:   QM/MM molecular dynamics simulations of Mn^2+^ and V^2+^. J. Am. Chem. Soc. **125**, 1618–1624 (2003). <https://doi.org/10.1021/ja0286831>
5. W.R. Cannon, B.M. Pettitt, J.A. McCammon, Sulfate anion in water: model structural, thermodynamic, and dynamic properties. J. Phys. Chem. **98**, 6225–6230 (1994). <https://doi.org/10.1021/j100075a027>
6. B. Golub, D. Ondo, R. Ludwig, D. Paschek, Why do liquids mix? the mixing of protic ionic liquids sharing the same cation is apparently driven by enthalpy, not entropy. J. Phys. Chem. Lett. **13**, 3556–3561 (2022). <https://doi.org/10.1021/acs.jpclett.2c00634>
7. B. Kaewruksa, A. Du, V. Ruangpornvisuti, Adsorption ability of pristine C24N24 nanocage promising as high hydrogen storage material: a DFT-D3 investigation. Int. J. Hydrog. Energy **47**, 29896–29906 (2022). <https://doi.org/10.1016/j.ijhydene.2022.06.286>
8. H. Li, Y. Chang, W. Zhu, W. Jiang, M. Zhang et al., A DFT study of the extractive desulfurization mechanism by[BMIM](+)[AlCl_4_](-) ionic liquid. J. Phys. Chem. B **119**, 5995–6009 (2015). <https://doi.org/10.1021/acs.jpcb.5b00516>
9. A.V. Marenich, C.J. Cramer, D.G. Truhlar, Performance of SM6, SM8, and SMD on the SAMPL1 test set for the prediction of small-molecule solvation free energies. J. Phys. Chem. B **113**, 4538–4543 (2009). <https://doi.org/10.1021/jp809094y>
10. X. Wang, J. Meng, X. Lin, Y. Yang, S. Zhou et al., Stable zinc metal anodes with textured crystal faces and functional zinc compound coatings. Adv. Funct. Mater. **31**, 2106114 (2021). <https://doi.org/10.1002/adfm.202106114>
11. Y. Yan, Y. Chu, M.A. Khan, M. Xia, M. Shi et al., Facile immobilization of ethylenediamine tetramethylene-phosphonic acid into UiO-66 for toxic divalent heavy metal ions removal: an experimental and theoretical exploration. Sci. Total Environ. **806**, 150652 (2022). <https://doi.org/10.1016/j.scitotenv.2021.150652>
12. Y. Jia, Y. Zhang, R. Wang, J. Yi, X. Feng et al., Mesoporous zirconium phosphonate hybrid material as adsorbent to heavy metal ions. Ind. Eng. Chem. Res. **51**, 12266–12273 (2012). <https://doi.org/10.1021/ie300253z>
13. C. Chang, S. Hu, T. Li, F. Zeng, D. Wang et al., A robust gradient solid electrolyte interphase enables fast Zn dissolution and deposition dynamics. Energy Environ. Sci. **17**, 680–694 (2024). <https://doi.org/10.1039/D3EE03422D>
14. T. Li, S. Hu, C. Wang, D. Wang, M. Xu et al., Engineering fluorine-rich double protective layer on Zn anode for highly reversible aqueous zinc-ion batteries. Angew. Chem. Int. Ed. **62**, e202314883 (2023). <https://doi.org/10.1002/anie.202314883>
15. S. Wu, H. Guo, Z. Su, C. Jia, X. Zhang et al., Suppressed manganese oxides shuttling in acidic electrolytes extends shelf-life of electrolytic proton batteries. Adv. Funct. Mater. **34**, 2315706 (2024). <https://doi.org/10.1002/adfm.202315706>
16. D. Chao, W. Zhou, C. Ye, Q. Zhang, Y. Chen et al., An electrolytic Zn–MnO_2_ battery for high-voltage and scalable energy storage. Angew. Chem. Int. Ed. **131**, 7905–7910 (2019). <https://doi.org/10.1002/ange.201904174>
17. A. Abdessemed, K.E. Djebbar, A.S. El-Kalliny, T. Sehili, H. Nugteren et al., Water treatment combined chlorine (monochloramine) degradation using direct photolysis and homogeneous photocatalysis (UV/H_2_O_2_, UV/NaOCl) with a medium pressure (MP) lamp as a source of UV. Int. J. Chem. React. Eng. **12**, 671–681 (2014). <https://doi.org/10.1515/ijcre-2014-0013>
18. M.K.S. Monteiro, Á. Moratalla, C. Sáez, E.V. Dos Santos, M.A. Rodrigo, Production of chlorine dioxide using hydrogen peroxide and chlorates. Catalysts **11**, 1478 (2021). <https://doi.org/10.3390/catal11121478>
19. G. Liang, B. Liang, A. Chen, J. Zhu, Q. Li et al., Development of rechargeable high-energy hybrid zinc-iodine aqueous batteries exploiting reversible chlorine-based redox reaction. Nat. Commun. **14**, 1856 (2023). <https://doi.org/10.1038/s41467-023-37565-y>
20. S. Yang, C. Li, H. Lv, X. Guo, Y. Wang et al., High-rate aqueous aluminum-ion batteries enabled by confined iodine conversion chemistry. Small Methods **5**, e2100611 (2021). <https://doi.org/10.1002/smtd.202100611>
21. P. Li, X. Li, Y. Guo, A. Chen, R. Zhang et al., Development of an energy-dense and high-power Li-Cl_2_ battery using reversible interhalogen bonds. Chem **10**, 352–364 (2024). <https://doi.org/10.1016/j.chempr.2023.09.021>
22. Y. Zou, T. Liu, Q. Du, Y. Li, H. Yi et al., A four-electron Zn-I2 aqueous battery enabled by reversible I−/I2/I+ conversion. Nat. Commun. **12**, 170 (2021). <https://doi.org/10.1038/s41467-020-20331-9>
23. X. Li, M. Li, Z. Huang, G. Liang, Z. Chen et al., Activating the I^0^/I^+^ redox couple in an aqueous I_2_–Zn battery to achieve a high voltage plateau. Energy Environ. Sci. **14**, 407–413 (2021). <https://doi.org/10.1039/d0ee03086d>
24. Y. Zhang, C. Wei, M.-X. Wu, Y. Wang, H. Jiang et al., A high-performance COF-based aqueous zinc-bromine battery. Chem. Eng. J. **451**, 138915 (2023). <https://doi.org/10.1016/j.cej.2022.138915>
25. D. Kundu, B.D. Adams, V. Duffort, S.H. Vajargah, L.F. Nazar, A high-capacity and long-life aqueous rechargeable zinc battery using a metal oxide intercalation cathode. Nat. Energy **1**, 16119 (2016). <https://doi.org/10.1038/nenergy.2016.119>
26. H. Pan, Y. Shao, P. Yan, Y. Cheng, K.S. Han et al., Reversible aqueous zinc/manganese oxide energy storage from conversion reactions. Nat. Energy **1**, 16039 (2016). <https://doi.org/10.1038/nenergy.2016.39>
27. X. Shi, Y. Sun, Y. Weng, X. Long, T. Lei et al., operando chemical strain analysis of CNT/VOOH during zinc insertion in Zn-ion batteries. Energy Environ. Sci. **16**, 4670–4678 (2023). <https://doi.org/10.1039/D3EE01745A>
28. J. Xie, Z. Liang, Y.-C. Lu, Molecular crowding electrolytes for high-voltage aqueous batteries. Nat. Mater. **19**, 1006–1011 (2020). <https://doi.org/10.1038/s41563-020-0667-y>
29. S. Cai, X. Chu, C. Liu, H. Lai, H. Chen et al., Water-salt oligomers enable supersoluble electrolytes for high-performance aqueous batteries. Adv. Mater. **33**, e2007470 (2021). <https://doi.org/10.1002/adma.202007470>
30. Z. Zhang, B. Xi, X. Wang, X. Ma, W. Chen et al., Oxygen defects engineering of VO_2_·xH_2_O nanosheets *via* *in situ* polypyrrole polymerization for efficient aqueous zinc ion storage. Adv. Funct. Mater. **31**, 2103070 (2021). <https://doi.org/10.1002/adfm.202103070>
31. M. Liu, Y. Zhang, Z. Xu, X. Han, W. Gou et al., Highly microporous carbon from *Enteromorpha* for high-performance aqueous zinc-chalcogen (S, SeS_2_) batteries. Batter. Supercaps **6**, e202300145 (2023). <https://doi.org/10.1002/batt.202300145>
32. C. Wei, Y. Wang, Z. Ding, T. Fang, J. Song et al., A universal strategy toward low-cost aqueous sulfur–iodine batteries. Adv. Funct. Mater. **33**, 2212644 (2023). <https://doi.org/10.1002/adfm.202212644>
